# Decoding cellular communication networks and signaling pathways in bone, skeletal muscle, and bone-muscle crosstalk through spatial transcriptomics in a young male mouse

**DOI:** 10.1038/s41413-026-00520-w

**Published:** 2026-05-19

**Authors:** Chuan Qiu, Yisu Li, Yun Gong, William Sherman, Di Tian, Weiqiang Lin, Zehui Pan, Boluwatife Afolabi, Vivek Thumbigere, Kuanjui Su, Jeffrey Deng, Yuwei Hou, Shashank Mungasavalli Gnanesh, Zhe Luo, Qing Tian, Fernando Sanchez, Yiping Chen, Hui Shen, Hong-Wen Deng

**Affiliations:** 1 Tulane Center for Biomedical Informatics and Genomics, Deming Department of Medicine, School of Medicine, Tulane University, New Orleans, LA USA; 2 Department of Cell and Molecular Biology, School of Science and Engineering, Tulane University, New Orleans, LA USA; 3 Department of Orthopedic Surgery, School of Medicine, Tulane University, New Orleans, LA USA; 4 Department of Pathology and Laboratory Medicine, School of Medicine, Tulane University, New Orleans, LA USA; 5 Division of Periodontology, Department of Advanced Oral Sciences & Therapeutics, School of Dentistry, University of Maryland, Baltimore, MD USA; 6 Geisel School of Medicine, Dartmouth College, Hanover, NH USA

**Keywords:** Metabolomics, Bone

## Abstract

Bone and skeletal muscle are essential components of musculoskeletal system, enabling movement, load-bearing, and systemic homeostasis. These tissues communicate through dynamic bone-muscle crosstalk mediated by cytokines, growth factors, and extracellular-matrix (ECM) proteins. The spatial organization of these mediators is critical for maintaining tissue integrity, and its disruption contributes to diseases, such as osteoporosis, sarcopenia, and metabolic syndrome. Despite this importance, spatial transcriptomics (ST) studies of bone-muscle interactions remain limited. Here, we applied 10x Genomics Visium ST with computational tools, e.g., SMART and CellChat, to deconvolute cell-type composition and characterize cell-cell communication networks and ligand-receptor (L-R) interactions in mouse femur and adjacent skeletal muscle. We identified eight major cell types (erythroid cells, endothelial cells, skeletal muscle cells, osteoblasts, myeloid cells, monocytes/macrophages, mesenchymal stem cells, and adipocytes) with distinct spatial transcriptional profiles and thirteen CellChat-inferred pathways, such as ECM-receptor related (e.g., COLLAGEN, TENASCIN, THBS) and secreted-signaling involved (e.g., VEGF) pathways. Representative L-R pairs include Col1a1/Col1a2-Sdc4, mediating osteoblast-to-muscle interactions, and Col4a1-Sdc4, facilitating muscle-to-osteoblast interactions in COLLAGEN, Tnxb-Sdc4 in TENASCIN, supporting muscle-to-osteoblast/muscle/myeloid/endothelial communication, Comp-Sdc4 in THBS, driving monocyte/macrophage-to-osteoblast/muscle signaling, and Vegfa-Vegfr1/Vegfr2 in VEGF, mediating muscle-to-endothelial/myeloid signaling. Immunostaining validated colocalization of several representative L-R pairs with their corresponding cells. Additionally, independent mouse and human bone scRNA-seq datasets reproduced most of the pathways and L-R pairs identified in ST, underscoring the robustness and cross-species relevance of our findings. Together, we present an initial spatially resolved transcriptome-wide map of bone-muscle intercellular communication, providing novel insights into molecular crosstalk and establishing groundwork for future studies in musculoskeletal disorders.

## Introduction

Bone and skeletal muscle are integral components of the musculoskeletal system, functioning not only as structural elements essential for movement and load-bearing but also as key regulators of systemic homeostasis.^1^ Their dynamic interplay, known as bone-muscle crosstalk, involves a complex network of biochemical signals mediated by cytokines, growth factors, and extracellular-matrix (ECM) proteins, alongside mechanical interactions.^2^ This intricate communication is essential for maintaining tissue homeostasis, adapting to mechanical stress, and facilitating repair after injury.^3,4^ Dysregulation of these processes contributes to musculoskeletal and metabolic disorders, such as osteoporosis, sarcopenia, and metabolic syndrome.^3–7^ Recent studies highlighted the molecular interdependence of bone and muscle.^8–12^ For example, bone-derived factors, such as osteocalcin, regulate muscle metabolism,^10^ while muscle-secreted myokines, such as irisin, influence bone remodeling and systemic energy expenditure.^11^ Additionally, many signaling pathways play pivotal roles in physiology of both tissues.^12–15^ For instance, collagen-involved pathways support tensile strength, ECM organization, and osteoblast/osteoclast activity,^12,13^ whereas Spp1-associated pathways contribute to bone remodeling, hematopoietic niche maintenance, and muscle regeneration.^14,15^

Although single-cell RNA sequencing (scRNA-seq) has significantly enhanced our understanding of cellular heterogeneity in musculoskeletal tissues,^16–22^ it lacks spatial context, limiting the ability to contextualize cellular changes within their native microenvironments or to map spatially dependent intercellular interactions. Spatial transcriptomics (ST) overcomes this limitation by integrating high-throughput gene expression profiling with in situ localization, enabling investigation of how cellular behavior is influenced by tissue architecture and intercellular communication.^23–25^ Recent studies have shown that the spatial organization of bone and muscle plays a critical role in regulating their structure and function.^26–32^ For example, integrated transcriptomic and imaging analyses have revealed region-specific gene expression and metabolic zonation within skeletal muscle.^26^ Similarly, combined ST and scRNA-seq profiling has identified spatial gradients of skeletal stem and progenitor cells and uncovered localized morphogenetic and metabolic programs in bone marrow.^27^ However, spatially resolved transcriptomic studies of bone-muscle interactions remain underexplored, leaving a critical gap in understanding the spatial organization and regulation of these interactions within tissue microenvironment.

In this study, we performed ST profiling of mouse femur bone and adjacent skeletal muscle, characterized cell-cell communication networks, and delineated ligand-receptor (L-R) interactions through signaling pathways. We validated the spatial colocalization of representative L-R pairs across various cell types using multiplex immunostaining. Independent mouse and human bone scRNA-seq datasets reproduced most signaling pathways and L-R pairs identified by ST, underscoring the robustness of our findings. Together, this work establishes an initial spatially resolved, transcriptome-wide map of bone-muscle intercellular communication and provides novel insights into the molecular mechanisms underlying musculoskeletal biology.

## Results

### Spatial transcriptomic profiling and cell type deconvolution of bone and skeletal muscle

**Fig. 1:**
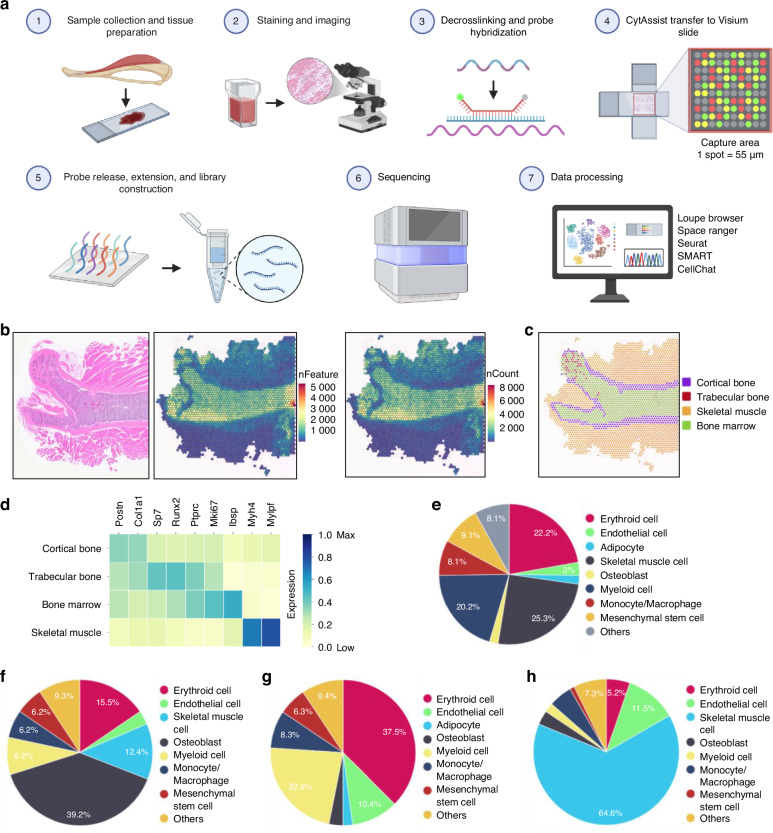
Spatial transcriptomic profiling of femur and adjacent skeletal muscle. **a** Schematic overview of the experimental design and workflow using the 10x Genomics Visium CytAssist Spatial Transcriptomics platform. (1) The femur and adjacent skeletal muscle from a 5-week-old male C57BL/6 mouse was harvested, fixed in 10% buffered formalin, decalcified, and paraffin-embedded. Tissue was sectioned at 4 μm thickness. (2) Section was stained with H&E for histology-guided annotation and spatial transcriptomic analysis. (3) After decrosslinking, probe hybridization with the Mouse Transcriptome Probe Set v1.0 was performed. (4) The user-supplied slide was aligned with a Visium CytAssist slide containing a 6.5 mm × 6.5 mm capture area with ~5 000 barcoded spots (55 μm diameter). (5) Probes hybridized to mRNA were ligated, released, and extended to incorporate unique spatial barcodes, followed by library construction. (6) Libraries were sequenced on the Illumina NextSeq 2000. (7) Data were analyzed using Loupe Browser, Space Ranger, Seurat, SMART, and CellChat etc. **b** H&E-stained section overlaid with spatial plots of detected genes (nFeature) and transcripts (nCount) per spot. **c** Spatial spot manual annotations in Loupe Browser v5.0.1 (purple: cortical bone; red: trabecular bone; green: bone marrow; yellow: skeletal muscle). **d** Gene expression pattern of representative marker genes within each manually annotated compartment. The right-side color bar shows relative gene expression level, scaled from 0 to 1 per gene across all spots; higher intensity indicates higher relative gene expression level. **e** SMART-predicted overall cell type composition across femur and skeletal muscle. SMART-predicted cell type composition within distinct tissue compartments: **f** cortical and trabecular bone, **g** bone marrow, and **h** skeletal muscle tissue

We performed ST analysis using 10x Genomics Visium on femur and adjacent skeletal muscle from a 5-week-old male C57BL/6 mouse (Fig. 1a). A total of 2 660 spatial spots (~55 µm diameter) were obtained, with a median of 506 unique genes per spot, encompassing 19 199 genes (Table 1, Fig. 1b). To evaluate spatially distinct expression patterns, the tissue was manually annotated into cortical bone, trabecular bone, bone marrow, and skeletal muscle based on H&E-registered image using Loupe Browser v5.0.1 (Fig. 1c). Some boundary spots assigned to a compartment may also contain cells from adjacent regions due to partial overlaps (e.g., trabecular bone spot overlapping bone marrow). The expression of representative compartment markers confirmed the accuracy of histology-guided segmentation (Fig. 1d). For example, osteogenic/osteoprogenitor genes (Col1a1, Sp7) were highly expressed in cortical/trabecular bones, whereas proliferative (Mki67) and hematopoietic (Ptprc) markers were predominant in bone marrow, consistent with prior findings.^27^ Notably, we observed elevated expression of myosin genes (Myh4, Mylpf) in skeletal muscle. Gene compartmental enrichment analysis further validated that these markers were specifically up-regulated within their respective compartments (Table S1). These findings demonstrate the feasibility of ST for mouse musculoskeletal tissues and provide a spatially resolved molecular map of bone and muscle.

**Table 1:** The 10x Genomics Visium transcriptomics data summary

Spots	Number
Number of spots under tissue	2 660
Mean reads per spot	105 186
Median UMI counts per spot	818
Median genes per spot	506
Genes detected	19 199

The high cellular heterogeneity of bone and muscle, along with the limited resolution of Visium platforms that capture transcripts from multiple neighboring cells per spot rather than single-cell profiles,^33–35^ poses challenges for accurate cell-type characterization. To address this, various deconvolution algorithms (e.g., CARD,^36^ Cell2location,^37^ and SMART^38^) have been developed and successfully applied across tissues (e.g., brain,^39,40^ kidney,^41^ and tumor microenvironments^42,43^), revealing fine-grained cellular architectures and spatially restricted signaling niches previously unresolved. Here, we employed SMART,^38^ which integrates ST data with curated cell-type markers to infer both cell-type-specific expression and cellular composition of each spot. Marker genes were curated from the Mouse Cell Atlas^44^ and CellMarker 2.0,^45^ ensuring a comprehensive and biologically relevant marker panel. We deconvolved eight major cell types, including erythroid cells, endothelial cells, skeletal muscle cells, osteoblasts, myeloid cells, monocytes/macrophages, mesenchymal stem cells (MSCs), and adipocytes, each comprising >2% of total cellular composition (Fig. 1e). Of the 2 660 mapped spots, 2 134 were dominated by a single cell type, while 526 mixed-profile spots (see “Materials and methods”) were primarily localized to boundary regions, such as cortical bone-marrow interfaces. As expected, osteoblasts were predominant in cortical and trabecular bone, reflecting their critical role in bone remodeling and ECM production. MSCs were also enriched in these regions, consistent with their contribution to osteogenic progenitor activity (Fig. 1f). In bone marrow, hematopoietic cells, including erythroid and myeloid cells, as well as endothelial cells were dominant, in line with their roles in hematopoietic and vascular niches (Fig. 1g). Skeletal muscle cells were confined to muscle regions, with minor infiltration of immune cells, such as monocytes/macrophages (Fig. 1h). To characterize tissue-specific transcriptional differences between bone and skeletal muscle, we performed differential gene expression and Gene Ontology (GO) analyses. Notably, we observed significant enrichment in GO terms related to muscle contraction and collagen metabolism (Table 2), reflecting the distinct functional roles of these tissues. Collectively, these results delineated the intricate cellular architecture of bone and skeletal muscle, providing groundwork for exploration of cell-cell communication within these interconnected systems.

**Table 2:** Gene Ontology analysis of differentially expressed genes identified through comparative analysis between bone and skeletal muscle

Enriched GO Terms	*P*-value
Striated muscle contraction	3.98E-08
Collagen chain trimerization	1.92E-05
Defective VWF binding to collagen type I	5.38E-05
Collagen degradation	1.82E-04
Assembly of collagen fibrils and other multimeric structures	1.90E-04
Enhanced binding of GP1BA variant to VWF multimer: collagen	2.90E-04
Defective binding of VWF variant to GPIb: IX: V	2.90E-04
Defects of platelet adhesion to exposed collagen	3.79E-04
Non-integrin membrane-ECM interactions	6.78E-04
MET activates PTK2 signaling	9.50E-04
ECM proteoglycans	1.20E-03
Anchoring fibril formation	1.31E-03
Degradation of the extracellular matrix	1.37E-03
Apoptosis induced DNA fragmentation	1.67E-03
RUNX3 regulates immune response and cell migration	2.08E-03
Collagen biosynthesis and modifying enzymes	3.20E-03
Crosslinking of collagen fibrils	3.29E-03
Collagen formation	6.64E-03
Scavenging by class a receptors	1.29E-02
DNA damage recognition in GG-NER	1.78E-02
RUNX2 regulates osteoblast differentiation	1.79E-02
NCAM1 interactions	2.59E-02
Integrin cell surface interactions	2.81E-02
RUNX2 regulates bone development	2.93E-02

A Bonferroni-corrected *P* < 0.05 was employed to define statistically significant enrichment terms

### Cell-cell communication networks in bone and skeletal muscle

Elucidating cell-cell communication is critical for understanding tissue function, as intercellular signaling orchestrates processes, such as immune regulation, stem cell maintenance, and tissue remodeling.^46–48^ Computational tools, e.g., CellChat^49^ and CellPhoneDB,^50^ reconstruct signaling networks, quantify sender-receiver relationships, and cluster pathways into functional modules.^49,50^ Incorporating spatial proximity further improves predictions by minimizing biologically implausible long-range interactions.^49^ These approaches have uncovered essential signals that maintain tissue homeostasis and drive pathological remodeling.^51–53^

**Fig. 2:**
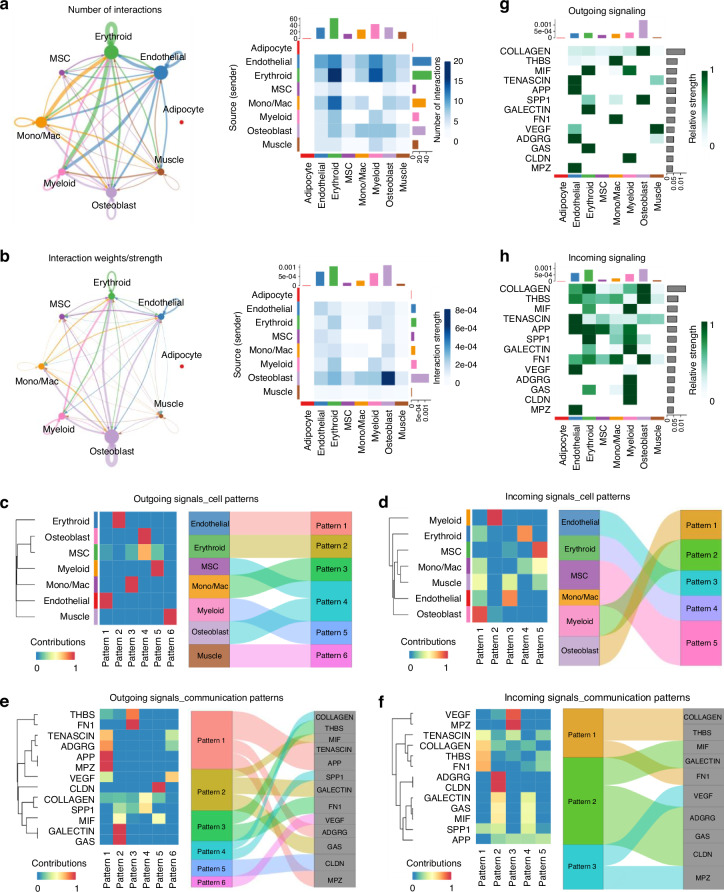
The cell-cell communication patterns within femur and adjacent skeletal muscle. **a** Chord diagram (left) depicting the number of inferred interactions between each pair of cell populations. Heatmap (right) showing interaction counts between sending (rows) and receiving (columns) cell population. **b** Chord diagram (left) depicting the strength (weight) of inferred interactions between each pair of cell populations. Heatmap (right) showing the weighted communication probability between senders and receivers. Higher intensity indicates a greater number of interactions (**a**) or stronger interactions (**b**) between the sender and the receiver. The numbers and strengths of interactions were computed by CellChat based on probabilistic modeling of L-R expression, with significance determined by permutation testing (*P* < 0.05). Outgoing (**c**) and incoming (**d**) signaling patterns by cell cluster (left) and enriched cell clusters with contribution >0.5 (right). Outgoing (**e**) and incoming (**f**) signaling patterns by pathway (left) and enriched pathway with contribution >0.5 (right). The “Contributions” (probability range 0–1) in **c**–**f** (left), which indicate the strength of alignment with each pattern, derives from non-negative matrix factorization (NMF) of communication probability matrices (mass-action L-R model). For the alluvial plots in **c**–**f** (right), the loading values < 0.5 are set to zero following CellChat convention to highlight enriched cell clusters or pathways. Heatmaps showing pathways that contribute to outgoing (**g**) and incoming (**h**) signals for each cell group. The “Relative strength” scale (0–1) reflects row-scaled normalization for each pathway across all cell groups. The top-colored bar: total strength of each cell group. The right gray bar: total strength of each pathway

To investigate intercellular communication in bone and muscle, we employed CellChat,^49^ enabling high-precision mapping of cell-cell signaling networks. Our analysis revealed extensive communications within femur and skeletal muscle (Fig. 2a, b). Erythroid cells, endothelial cells, and monocytes/macrophages exhibited the highest levels of communication (>10 interactions), forming dense networks with most other cell types (Fig. 2a). The largest number of interactions was observed between endothelial and erythroid/myeloid cells, erythroid and myeloid cells, as well as monocytes/macrophages and erythroid cells. Intriguingly, erythroid cells displayed robust autocrine signaling. Osteoblasts, while displaying a moderate number of intercellular communications, showed extensive interactions with all other cell types, including robust autocrine loop (Fig. 2b). Skeletal muscle cells exhibited moderate intercellular communication with endothelial, erythroid, monocyte/macrophage, and osteoblast populations (Fig. 2a, b), indicating relatively weak communication between skeletal muscle and bone. In this study, we did not identify significant adipocyte-mediated communication (Fig. 2a, b), which is likely due to both biological and/or technical reasons. Biologically, bone tissue from a healthy young male C57BL/6 mouse naturally exhibits low marrow adiposity.^54^ Technically, adipocytes are challenging to be captured in ST because of their fragility and limited RNA recovery during tissue processing. Their low representation also reduces statistical power in CellChat, resulting in under-calling of cell-cell communications. Therefore, the absence of adipocyte signaling in this dataset should not be interpreted as a lack of biological involvement in musculoskeletal tissues. Further studies are warranted to elucidate their functional roles in these contexts.

Next, we characterized outgoing and incoming signaling patterns in femur and skeletal muscle and identified 6 outgoing patterns, reflecting dominant ligand-driven communication programs from specific cell populations, and 5 incoming patterns, representing receptor-mediated response signatures shared across multiple cell types (Fig. 2c–f). For outgoing signals, patterns 1, 3, 4, and 5 were primarily driven by endothelial cells, monocytes/macrophages, osteoblasts, and myeloid cells, respectively. Notably, pattern 4 also demonstrated significant involvement of MSCs. In contrast, patterns 2 and 6 were predominantly restricted to erythroid and skeletal muscle cells, respectively (Fig. 2c). For incoming signals, each pattern was shared across multiple cell clusters, indicating a more intricate and interconnected communication network compared to outgoing signaling patterns (Fig. 2d). To further elucidate the molecular mechanisms underlying these communication patterns, we performed pathway analysis and identified 13 CellChat-inferred signaling pathways spanning ECM-receptor related pathways (COLLAGEN, SPP1, THBS, FN1, TENASCIN), secreted-signaling involved pathways (VEGF, MIF, GALECTIN, GAS), and other pathways (ADGRG, APP, CLDN, MPZ). Each pathway was enriched in distinct communication patterns (Fig. 2e–f). For the outgoing signals, TENASCIN, ADGRG, APP, and MPZ strongly contributed to pattern 1, GALECTIN, GAS, and MIF dominated pattern 2, THBS and FN1 played critical roles in pattern 3, COLLAGEN and SPP1 were key in pattern 4, and CLDN and VEGF were associated with patterns 5 and 6, respectively (Fig. 2e). Similarly, for the incoming signals, COLLAGEN, THBS, and FN1 strongly contributed to pattern 1, ADGRG, CLDN, GALECTIN, GAS, and MIF were enriched in pattern 2, and VEGF and MPZ dominate pattern 3 (Fig. 2f). The contribution of these signaling pathways to intercellular communication in various cell clusters was illustrated in Fig. 2g, h. For example, both outgoing and incoming signals in osteoblasts were inferred to be mediated by COLLAGEN and SPP1 signaling pathways, which are consistent with previously reported roles in orchestrating bone matrix production, mineralization, and the structural integrity of bone tissue.^12,14^ Outgoing signals in skeletal muscle cells were driven by VEGF and TENASCIN, which regulate angiogenesis, vascular niche maintenance, ECM remodeling, and muscle repair.^55–60^ Monocytes/macrophages exhibited strong outgoing signals via THBS and FN1, with osteoblasts being dominant receivers (Fig. 2g, h). These results provided novel insights into the molecular mechanisms that may drive bone-muscle crosstalk.

### The role of L-R interactions in COLLAGEN and SPP1 signaling pathways regulating osteoblast function

**Fig. 3:**
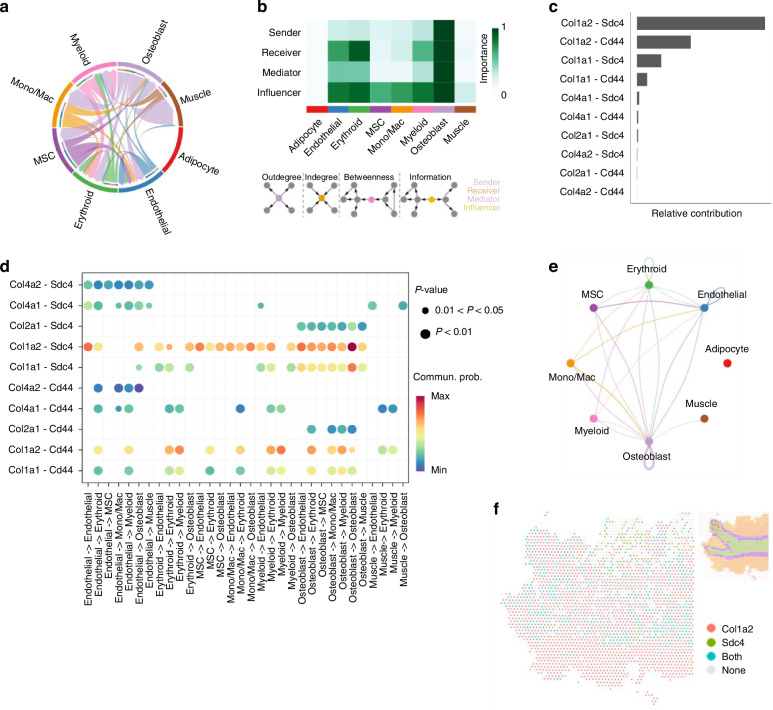
Cell-cell communications and their corresponding L-R interaction pairs in COLLAGEN pathway. **a** A chord diagram showing cell-cell communications in COLLAGEN pathway. **b** Network centrality of the COLLAGEN signaling pathway. The importance of each cell cluster was evaluated using network centrality measures derived from CellChat. Pathway-specific weighted directed graphs were constructed, with edge weights representing the summed communication probabilities of significant L-R pairs. Sender, receiver, mediator, and influencer scores correspond to normalized out-degree, in-degree, flow betweenness, and information centralities, respectively, reflecting each cell group’s signaling activity and influence. All values were scaled to (0, 1), with darker colors indicating greater importance. **c** Relative contributions of L-R pairs within the pathway. **d** Dot plot of significant L-R pairs across various cell types in COLLAGEN signaling pathway. For each L-R pair, color represents the communication probability, and dot size indicates the statistical significance of the interaction within each cell-cell communication pair. Communication probabilities were estimated using a mass-action model that integrates ligand, receptor, and cofactor expression levels to quantify relative interaction strength (scaled 0–1). Statistical significance (*P* < 0.05) was determined by permutation testing (*M* = 100) comparing observed probabilities to randomized cell-type labels. **e** Cell-cell communication mediated by Col1a2-Sdc4 L-R pair in COLLAGEN signaling pathway. **f** A diagram shows the spatial localization of cell-cell communications via Col1a2-Sdc4 L-R pair in COLLAGEN pathway

L-R interactions orchestrate bone and skeletal muscle microenvironment by regulating ECM remodeling, angiogenesis, and immune cell recruitment.^61–63^ Within this framework, osteoblasts emerged as central hubs in COLLAGEN and SPP1 signaling pathways, which regulate ECM remodeling, cell adhesion, bone remodeling, and hematopoietic niche maintenance.^64–68^ Their multifaceted roles in these pathways highlight their importance in sustaining musculoskeletal homeostasis and mediating bone-muscle crosstalk.^2,69–71^ In COLLAGEN pathway, we observed a sophisticated network of cell-cell interactions (Fig. 3a). Notably, osteoblasts functioned as dominant senders, receivers, mediators, and influencers, highlighting their critical role in COLLAGEN signaling pathway (Fig. 3b). We identified 10 significant L-R pairs (Fig. 3c, d, Fig. S1, Table S2), encompassing 5 ligand genes, including Col1a1 (collagen type I alpha 1 chain, the major fibrillar collagen subunit that pairs with Col1a2 to form type I collagen), Col1a2 (collagen type I alpha 2 chain, a fibrillar collagen subunit that combines with Col1a1 to form type I collagen), Col2a1 (collagen type II alpha 1 chain, the primary fibrillar collagen in cartilage ECM), Col4a1 (collagen type IV alpha 1 chain, a non-fibrillar collagen forming the basement membrane scaffold), and Col4a2 (collagen type IV alpha 2 chain, pairs with Col4a1 in basement membranes), and 2 receptor genes, including Sdc4 (syndecan-4, a transmembrane heparan sulfate proteoglycan receptor) and Cd44 (CD44, a cell-surface glycoprotein receptor for hyaluronic acid) in COLLAGEN signaling pathway. Among these, Col1a1/Col1a2-Sdc4 pairs emerged as dominant contributors, mediating extensive interactions across multiple cell clusters, such as outgoing signals from osteoblasts to most clusters and outgoing signals from erythroid and myeloid cells to endothelial cells, erythroid cells, and osteoblasts. Moreover, autocrine signaling within osteoblasts further highlighted the critical role of Col1a2-Sdc4 L-R pairs in COLLAGEN signaling pathway (Fig. 3e, f, Fig. S2b). Previous studies demonstrated that Sdc4 is essential for endochondral ossification and fracture repair,^72^ while its deletion disrupts matrix homeostasis and accelerates age-related osteopenia.^73^ Sdc4 deficiency also perturbs collagen balance, with increased Col1a1 expression in mutant mice.^74^ The SPP1 signaling pathway demonstrated a moderately complex interaction network (Fig. S3a). Erythroid cells and monocytes/macrophages were highly active as receivers, mediators, and influencers, while osteoblasts emerged as dominant senders and influencers (Fig. S3b). Osteoblasts interacted with erythroid cells, monocytes/macrophages and myeloid cells through the L-R pair, Spp1-Cd44 (Spp1: Osteopontin, a secreted phosphoprotein) (Fig. S3c, d, Table S2). Notably, robust autocrine signaling was observed in osteoblasts, monocytes/macrophages, and erythroid cells, emphasizing their regulatory roles through Spp1-Cd44 L-R pair in SPP1 signaling pathway (Fig. S3c, d). Interestingly, previous studies reported that Spp1 mediates cell adhesion and migration via Cd44 and integrins.^75,76^ Loss of Spp1 reduces Cd44 expression and impairs osteoclast function.^77^

### The role of L-R interactions in THBS and FN1 signaling pathways regulating monocyte/macrophage function

**Fig. 4:**
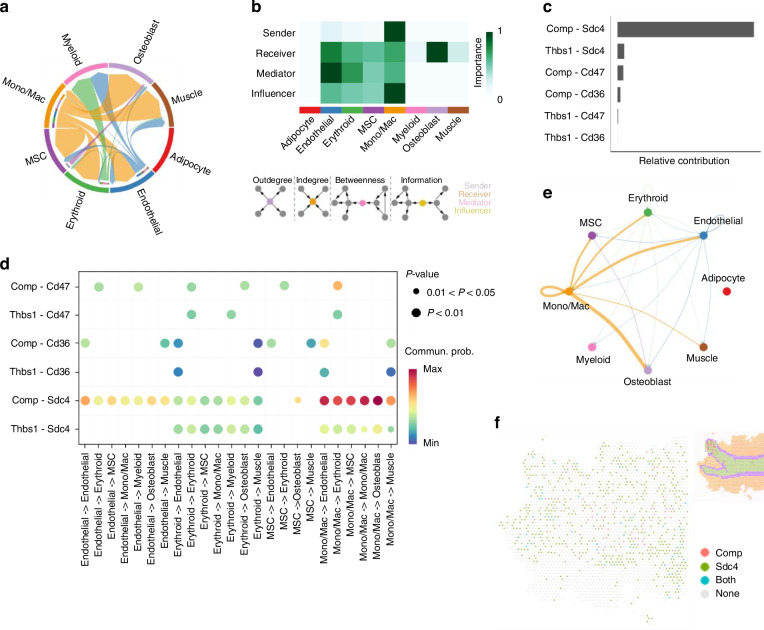
Cell-cell communications and their corresponding L-R interaction pairs in THBS pathway. **a** A chord diagram showing cell-cell communications in THBS pathway. **b** Network centrality of the THBS signaling pathway. The darker colors indicate greater importance. **c** Relative contributions of L-R pairs within the pathway. **d** Dot plot of significant L-R pairs across various cell types in THBS signaling pathway (color = communication probability; dot size = significance). **e** Cell-cell communication mediated by Comp-Sdc4 L-R pair in THBS signaling pathway. **f** A diagram shows the spatial localization of cell-cell communications via Comp-Sdc4 L-R pair in THBS pathway

Monocytes/macrophages play a central role in THBS and FN1 pathways, which regulate bone remodeling, ECM organization, stem cell niches, and muscle repair.^78–84^ Within THBS signaling pathway, we identified a highly interconnected network of cell-cell interactions (Fig. 4a). Most cell types demonstrated strong activity as receivers, mediators, and influencers, with monocytes/macrophages emerging as dominant senders, receivers, mediators, and influencers, whereas osteoblasts served primarily as receivers (Fig. 4b). Six significant L-R pairs were identified in THBS signaling pathway (Fig. 4c, d, Fig. S4, Table S2), involving 2 ligand genes, including Thbs1 (thrombospondin-1, a matricellular glycoprotein) and Comp (cartilage oligomeric matrix protein, a pentameric ECM glycoprotein), and 3 receptor genes, including Cd47 (integrin-associated protein, a cell-surface glycoprotein receptor), Cd36 (platelet glycoprotein 4, a scavenger receptor for thrombospondin-1, oxidized LDL, and long-chain fatty acids), and Sdc4. Notably, Comp-Sdc4 pair was a dominant contributor to cell-cell interactions, facilitating robust outgoing signals from monocytes/macrophages to osteoblasts, endothelial cells, MSCs, and skeletal muscle cells. We also observed outgoing signals from MSCs, erythroid cells, and endothelial cells to osteoblast, as well as outgoing signals from erythroid cells and endothelial cells to skeletal muscle cells (Fig. 4d–f, Fig. S2c). Comp is highly expressed in musculoskeletal tissues and has been implicated in osteoarthritis.^85^ Mutations in Comp disrupt bone integrity, joint function, and skeletal development, causing severe long bone shortening.^86^ FN1 signaling pathway exhibited a sparser interaction network (Fig. S5a). Although most cell types were active as receivers and influencers, monocytes/macrophages notably emerged as dominant senders, receivers, and influencers, with osteoblasts primarily functioning as receivers (Fig. S5b). We identified 2 significant L-R pairs in FN1 signaling pathway (Fig. S5c–e, Table S2), Fn1-Sdc4 (Fn1: Fibronectin 1, a high-molecular-weight glycoprotein of the ECM) and Fn1-Cd44, mediating robust outgoing signals from monocytes/macrophages to osteoblasts, endothelial cells, MSCs, erythroid cells, and skeletal muscle cells. Monocytes/macrophages also exhibited strong autocrine signaling, further emphasizing their multifunctional roles in orchestrating both THBS and FN1 signaling pathways. Prior studies have shown that moesin co-immunoprecipitated with both Cd44 and Fn1, and that loss of either disrupts this ECM-receptor interaction.^87^ Additionally, Fn1-Sdc4/Cd44 signaling contributes to altered cell adhesion and immune regulation in cervical cancer.^88^

### The role of L-R interactions in TENASCIN and VEGF signaling pathways regulating skeletal muscle cell function

**Fig. 5:**
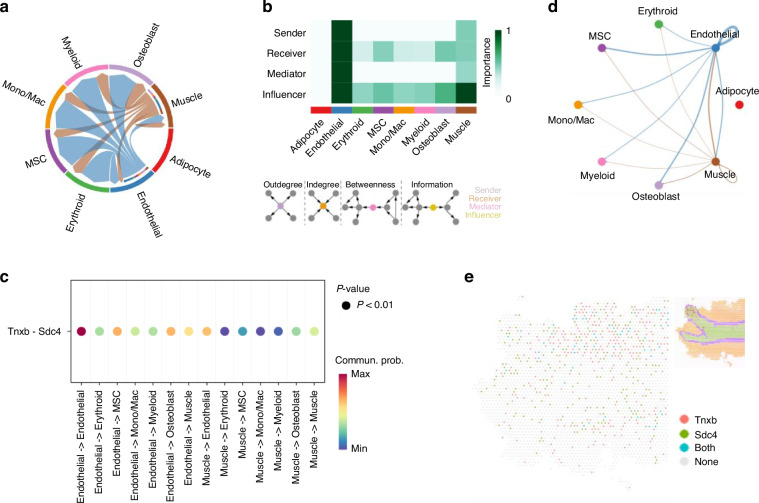
Cell-cell communications and their corresponding L-R interaction pairs in TENASCIN pathway. **a** A chord diagram showing cell-cell communications in TENASCIN pathway. **b** Network centrality of the TENASCIN signaling pathway. The darker colors indicate greater importance. **c** Dot plot of significant L-R pairs across various cell types in TENASCIN signaling pathway (color = communication probability; dot size = significance). **d** Cell-cell communication mediated by Tnxb-Sdc4 L-R pair in TENASCIN signaling pathway. **e** A diagram shows the spatial localization of cell-cell communications via Tnxb-Sdc4 L-R pair in TENASCIN pathway

Skeletal muscle cells actively engaged in TENASCIN and VEGF signaling pathways, which regulate angiogenesis, vascular niche maintenance, ECM remodeling, and muscle repair.^57,89,90^ In TENASCIN pathway, a moderate network of cell-cell interactions was identified (Fig. 5a). Skeletal muscle cells exhibited robust activity as influencers and moderate roles as senders and receivers, while endothelial cells demonstrated dominant roles as sender, receiver, mediator, and influencers (Fig. 5b). We identified a significant L-R pair in TENASCIN signaling pathway, comprising ligand gene Tnxb (Tenascin-X, a large ECM glycoprotein that regulates ECM assembly, collagen fibrillogenesis, and tissue elasticity) and receptor gene Sdc4 (Fig. 5c, Table S2). Interactions facilitated by Tnxb-Sdc4 pair revealed that skeletal muscle cells and endothelial cells communicate with each other and send outgoing signals to erythroid cells, MSCs, monocytes/macrophages, myeloid cells, and osteoblasts (Fig. 5c–e, Fig. S2d). Despite limited evidence for Tnxb-Sdc4 interaction, Tnxb maintains ECM integrity, and deficiency in Tnxb is associated with tissue fragility and musculoskeletal weakness.^91^ In bone, Tnxb restrains osteoclast multinucleation and resorption, with its loss leading to bone loss.^92^ The VEGF signaling pathway exhibited a relatively sparse interaction network (Fig. S6a). Within this pathway, skeletal muscle cells demonstrated strong activity as senders and influencers, while endothelial cells emerged as dominant receivers and influencers, reflecting their pivotal role in angiogenesis and vascular niche maintenance (Fig. S6b). We identified 2 significant L-R pairs in VEGF signaling pathway, Vegfa-Vegfr1 (Vegfa: Vascular endothelial growth factor A, a secreted growth factor that binds VEGFR1/VEGFR2; Vegfr1: Vascular endothelial growth factor receptor 1, a high-affinity receptor tyrosine kinase for VEGFA) and Vegfa-Vegfr2 (Vegfr2: Vascular endothelial growth factor receptor 2, the primary signaling receptor for VEGFA) (Fig. S6c, d, Table S2). Notably, Vegfa-Vegfr1 pair mediated interactions from skeletal muscle cells to endothelial cells (Fig. S6e), whereas Vegfa-Vegfr2 pair facilitated signaling to myeloid cells (Fig. S6f). Vegfa-Vegfr2 signaling has been shown to promote muscle regeneration and survival after injury,^93^ while in bone, Vegfa-Vegfr1/Vegfr2 coordinates angiogenesis and osteogenesis, and its inhibition impairs vascular invasion and bone regeneration.^94^

### The L-R pattern of bone-muscle crosstalk

**Fig. 6:**
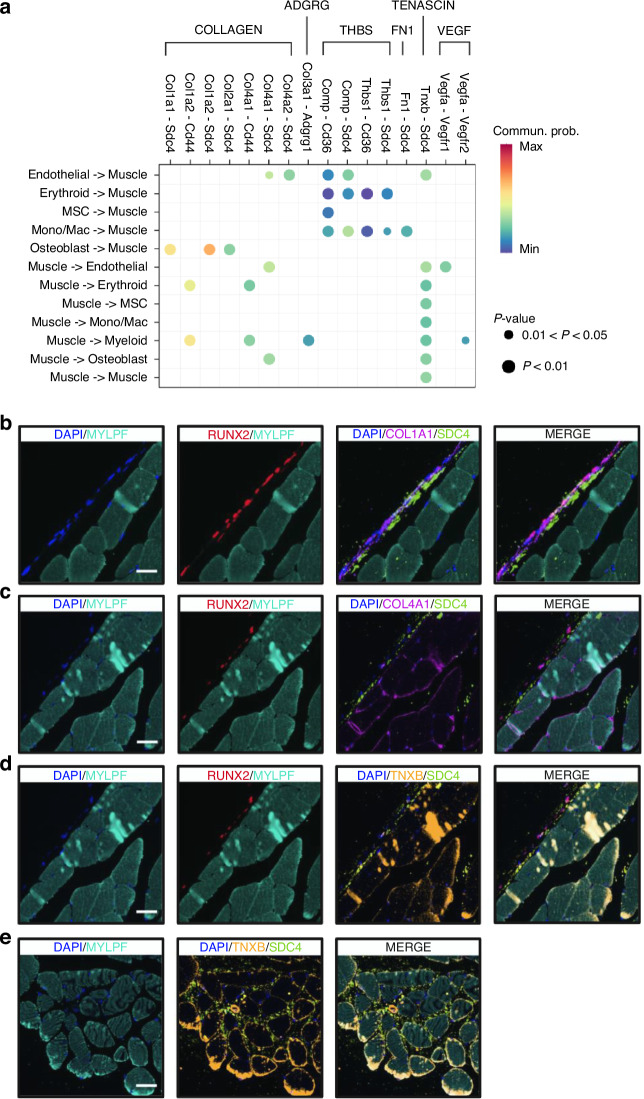
The L-R pattern of bone-muscle crosstalk. **a** A comprehensive map of L-R pairs through which skeletal muscle cells send signals to or receive signals from bone- and bone-marrow-enriched cell groups, potentially contributing to bone-muscle crosstalk. For each L-R pair, color reflects communication probability, and dot size indicates the statistical significance of the interaction within each cell-cell communication pair. **b**–**e** COMET multiplex immunostaining validation for the colocalization of representative L-R pairs with their corresponding cells: **b** The colocalization of osteoblast (Runx2^+^)-skeletal muscle cell (Mylpf^+^) through Col1a1-Sdc4 L-R pair. Scale bar, 20 µm. **c** The colocalization of skeletal muscle cell (Mylpf^+^)-osteoblast (Runx2^+^) through Col4a1-Sdc4 L-R pair. Scale bar, 40 µm. **d** The colocalization of skeletal muscle cell (Mylpf^+^)-osteoblast (Runx2^+^) through Tnxb-Sdc4 L-R pair. Scale bar, 40 µm. **e** The colocalization of skeletal muscle cell-skeletal muscle cell (Mylpf^+^) through Tnxb-Sdc4 L-R pair. Scale bar, 40 µm

To further elucidate bone-muscle crosstalk, we focused on L-R pairs in skeletal muscle cells that either transmit signals to or receive signals from cell populations enriched in bone or bone marrow (Fig. 6a). Beyond the L-R pairs described above, skeletal muscle cells also interact with erythroid and myeloid cells through Col1a2-Cd44 and Col4a1-Cd44 pairs, and influence osteoblast activity via Col4a1-Sdc4 pair, within COLLAGEN signaling pathway. Additionally, skeletal muscle cells communicate with myeloid cells via Col3a1-Adgrg1 pair in ADGRG signaling pathway. To validate these computationally predicted interactions at the protein level, we performed COMET multiplex immunostaining on tissue sections from the same mouse used in our Visium ST experiment (Fig. 6b–e). We confirmed the colocalization of osteoblasts (Runx2^+^) and skeletal muscle cells (Mylpf^+^) through Col1a1-Sdc4 (Fig. 6b), Col4a1-Sdc4 (Fig. 6c), and Tnxb-Sdc4 (Fig. 6d) L-R pairs. In addition, skeletal muscle-skeletal muscle interactions mediated by Tnxb-Sdc4 (Fig. 6e) were also validated. These results support the robustness of our computational inferences and underscore the functional relevance of these L-R interactions in mediating bone-muscle crosstalk.

### Partial validation of the L-R landscape using independent mouse and human bone scRNA-seq datasets

**Fig. 7:**
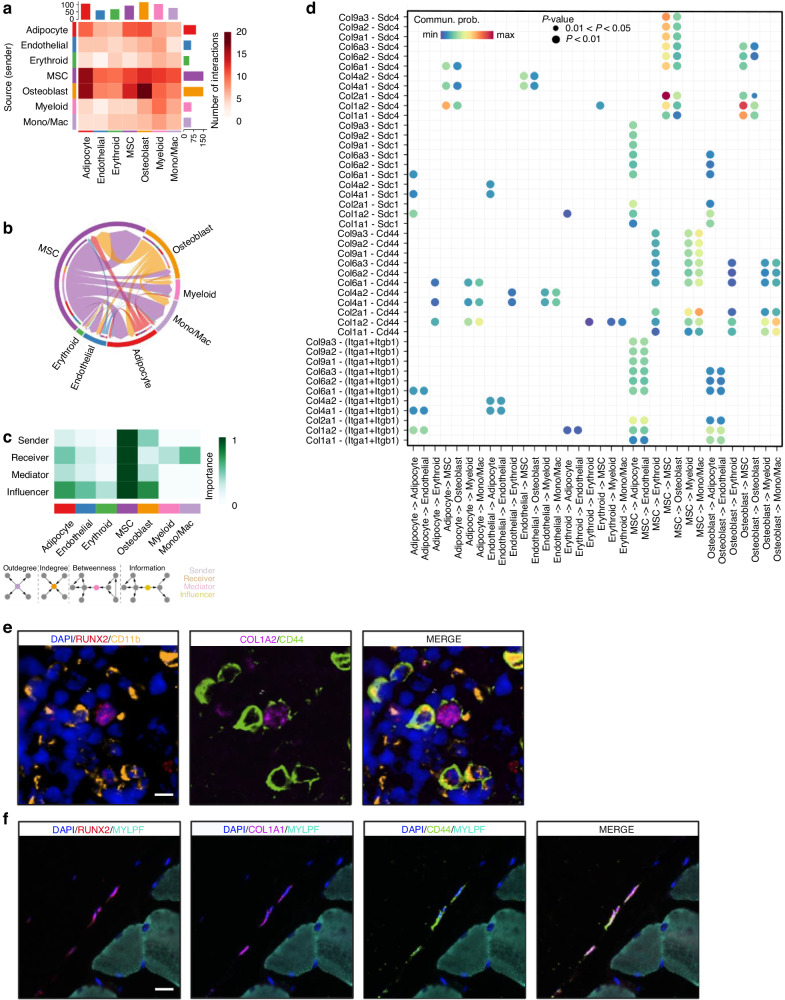
The cell-cell communication pattern in mice bone scRNA-seq dataset. **a** Heatmap showing interaction counts between sending (rows) and receiving (columns) cell population. **b** A chord diagram showing cell-cell communications in COLLAGEN pathway. **c** Network centrality roles computed on the pathway-specific weighted-directed network: senders (out-degree), receivers (in-degree), mediators (flow betweenness), influencers (information centrality). Darker color indicates greater role magnitude. **d** Dot plot of significant L-R pairs across various cell types in COLLAGEN signaling pathway. For each L-R pair, color represents the communication probability, and dot size indicates the statistical significance of the interaction within each cell-cell communication pair. **e**, **f** COMET multiplex immunostaining validation for the colocalization of representative L-R pairs with their corresponding cells: **e** The colocalization of osteoblast (Runx2^+^)-myeloid cell (Cd11b^+^) through Col1a2-Cd44 L-R pair. Scale bar, 10 µm. **f** The colocalization of osteoblast-osteoblast (Runx2^+^) through Col1a1-Cd44 L-R pair. Scale bar, 20 µm

To further evaluate the robustness and reproducibility of our ST-derived L-R interaction landscape, we performed partial validation using independent scRNA-seq datasets from both mouse and human bone/bone marrow. Although lacking skeletal muscle populations, and thus not directly capturing bone-muscle crosstalk, these datasets provide high-resolution, cell type-specific profiles of bone-resident populations that enable partial validation of ST-inferred communication networks. Using scRNA-seq data from femur and tibia bones of four 8–10-week-old male C57BL/6 mice,^95^ we identified a complex network of cell-cell interactions, including osteoblast-mediated communication with adipocyte, MSCs, and through autocrine signals (Fig. 7a). MSCs exhibited more interactions with other cell types, consistent with stromal cell enrichment in this dataset. Notably, 10 of the 13 signaling pathways identified in ST dataset were replicated in mouse bone scRNA-seq (Fig. S7). In COLLAGEN pathway, extensive communication was observed among MSCs, osteoblasts, and adipocytes (Fig. 7b), with MSCs acting as dominant senders, receivers, mediators, and influencers (Fig. 7c). We identified 44 significant L-R pairs involving 11 ligands and 4 receptors (Fig. 7d), including all 10 L-R pairs (e.g., Col1a2-Cd44 and Col1a1-Sdc4) identified in our ST dataset. Notably, multiplex immunostaining showed colocalization of osteoblast (Runx2^+^)-myeloid cell (CD11b^+^) through Col1a2-Cd44 interaction in bone marrow (Fig. 7e), and osteoblast-osteoblast colocalization mediated by Col1a1-Sdc4 signaling at bone-muscle interface (Fig. 6b). Immunostaining also confirmed osteoblast-osteoblast colocalization mediated by Col1a1-Cd44 within ST dataset (Fig. 7f). Although Col1a2-Sdc4 and Col1a2-Cd44 emerged as top contributors in both ST and scRNA-seq datasets, these interactions exhibited context-specific patterns. In mouse scRNA-seq datasets, for example, they primarily mediated signaling between adipocytes and MSCs, osteoblasts and MSCs, as well as osteoblasts and monocytes/macrophages. Such variation likely reflects differences in tissue composition, transcriptional states, and technical factors inherent to ST and scRNA-seq platforms. Beyond COLLAGEN pathway, we also examined intercellular communication patterns and validated the majority of L-R pairs within THBS (Fig. S8a–c), SPP1 (Fig. S8d–f), and FN1 (Fig. S8g–i) signaling pathways.

We further validated our findings using scRNA-seq data from three human femoral head samples. Complex communication was observed among osteoblasts, MSCs, endothelial cells, and adipocytes (Fig. S9a). As expected, interactions involving monocytes/macrophages were limited due to depletion of CD45^+^ cells during sample processing, while erythroid interactions were reduced, likely reflecting age- and disease-associated changes. Strikingly, all 13 pathways identified by ST were replicated in human scRNA-seq dataset (Fig. S9b). Although cell-cell communication patterns varied across pathways, most L-R pairs were consistently validated, including those in COLLAGEN (Fig. S10a–c), THBS (Fig. S10d–f), FN1 (Fig. S11a–c), SPP1 (Fig. S11d–f), TENASCIN (Fig. S12a–c), and VEGF (Fig. S12d–f). Interestingly, we identified strong MSC-to-adipocyte and osteoblast-to-adipocyte interactions mediated by (i) Col1a1/Col1a2/Col6a1/Col6a2/Col6a3-Sdc1 in COLLAGEN pathway (Fig. S10c), (ii) Thbs1-Sdc1 in THBS pathway (Fig. S10f), (iii) Fn1-Sdc1 in FN1 pathway (Fig. S11c), and (iv) Tnc-Sdc1 in TENASCIN pathway (Fig. S12c), indicating the potential role of MSC/osteoblast-adipocyte signaling in regulating marrow adiposity and tissue remodeling during aging and under osteoarthritic conditions. Together, these cross-platform analyses provide partial yet compelling validation of our ST-derived L-R landscape. The convergence of findings across mouse and human scRNA-seq datasets, coupled with protein-level confirmation of representative L-R pair colocalization, underscores the robustness of these signaling pathways and their L-R interactions in regulating intercellular communication in bone, skeletal muscle, and their crosstalk.

## Discussion

This study presents a spatially resolved, transcriptome-wide map of cell-cell interactions within bone and skeletal muscle microenvironments, highlighting intricate intercellular communication networks that sustain tissue homeostasis and bone-muscle crosstalk. By integrating ST with computational deconvolution, we systematically mapped L-R interactions across diverse cell types, offering exploratory insights into potential molecular mechanisms relevant to musculoskeletal health.

Our results demonstrated the feasibility of applying ST to bone and adjacent skeletal muscle, overcoming longstanding technical challenges in preserving and profiling these complex tissues. Rigorous quality control and distinct tissue-specific expression profiles underscore the robustness of our approach in delineating spatially resolved transcriptional landscapes. The inferred intercellular communication networks revealed coordinated interactions among diverse cell populations, particularly osteoblasts, monocytes/macrophages, skeletal muscle cells, and endothelial cells. Notably, within this dataset, osteoblasts and monocytes/macrophages appeared as prominent contributors to their respective signaling pathways, reflecting their multifaceted roles in tissue remodeling, immune regulation, and ECM organization. Robust autocrine signaling observed in osteoblasts, erythroid cells, and monocytes/macrophages underscores the regulatory feedback mechanisms intrinsic to these microenvironments. For example, erythroid cells regulate their differentiation, survival, and function through intrinsic mechanisms, including transcription factors, autocrine signaling, and metabolic controls.^96^

Furthermore, our findings provide exploratory evidence for the spatially organized cell-cell communication pattern in bone and skeletal muscle. Signaling pathways such as the TENASCIN and VEGF in skeletal muscle cells, THBS and FN1 in monocytes/macrophages, alongside COLLAGEN and SPP1 in osteoblasts, represent key mediators of these communications. Distinct outgoing and incoming signaling patterns highlight functional specialization of cell types in musculoskeletal tissues. For instance, skeletal muscle cells were prominent senders in the VEGF pathway, promoting angiogenesis and vascular remodeling,^57,89,90^ while osteoblasts exhibited strong outgoing signals in the COLLAGEN and SPP1 pathways, supporting bone remodeling and hematopoietic niche regulation.^64–68^ The analysis of L-R pairs in these key signaling pathways further offers insights into their functional roles. COLLAGEN pathway maintains structural integrity across bone, marrow, and muscle, regulating osteoblast/osteoclast activity, hematopoietic support, and muscle repair.^64–68,97–103^ Osteoblasts were dominant senders, with Col1a2-Sdc4 pair mediating extensive ECM interactions. The SPP1 pathway, crucial for bone remodeling and muscle regeneration,^104–106^ involved erythroid and myeloid cells as receivers and osteoblasts as key senders through Spp1-Cd44 signaling. The THBS pathway contributed to bone remodeling and stem cell niche maintenance,^78,80,107,108^ where monocytes/macrophages acted as major senders and osteoblasts as receivers, with Comp-Sdc4 facilitating broad intercellular communication. In FN1 pathway, monocytes/macrophages transmitted Fn1-Sdc4 and Fn1-Cd44 signals to osteoblasts and other cell types, reinforcing their role in ECM regulation.^84,109–111^ The VEGF pathway promoted angiogenesis,^93,112–115^ with skeletal muscle cells signaling to endothelial and myeloid cells via Vegfa-Vegfr1/2, highlighting vascular remodeling. Finally, the TENASCIN pathway supported ECM remodeling and bone-muscle crosstalk,^56,116–118^ with Tnxb-Sdc4 mediating interactions between skeletal muscle and endothelial cells. Dysregulation of these signaling pathways has been implicated in musculoskeletal and metabolic disorders, including osteoporosis, sarcopenia, and fibrosis.^3–7,27^ Our findings provide candidate targets for future studies of how these pathways and their L-R interactions are perturbed in pathological conditions.

Importantly, our validation analyses confirmed the robustness and biological relevance of the inferred intercellular signaling networks. Multiplex immunostaining verified key bone-muscle interactions, including Col1a1-Sdc4, Col4a1-Sdc4, and Tnxb-Sdc4 between osteoblasts and skeletal muscle cells, as well as Col1a2-Cd44 between osteoblasts and myeloid cells in bone marrow. Cross-platform validation using mouse and human bone scRNA-seq datasets reproduced most of the signaling pathways identified in ST (mouse: 10/13; human: 13/13) and confirmed numerous L-R pairs across COLLAGEN, THBS, FN1, SPP1, TENASCIN, and VEGF pathways.

Despite its novelty, this study has several limitations that inform directions for future improvement. First, partial bone detachment during Visium slide preparation, attributable to high density and mineralization of bone, may have affected spatial continuity and mapping accuracy, particularly in cortical bone of femoral head (Fig. S13). Minor misalignment or incomplete tissue transfers between H&E and Visium slides may also introduce subtle discrepancies between histological and ST images. Second, despite osteocytes are abundant and functionally critical, they were underrepresented in our study because their lacunar embedding within mineralized matrix and decalcification/permeabilization fragment RNA, yielding weak signals for osteocytes, as evidenced by osteocyte markers, such as Dmp1, Mepe, and Pdpn (Fig. S14). Spot-level mixing further dilutes sparse cortical osteocyte transcripts with signals from bone-surface and marrow compartments, limiting deconvolution robustness. Future work is needed to address these hurdles using hard-tissue-optimized protocols. Third, the Visium platform captures transcripts from multiple cells per spot, lacking single-cell resolution and potentially obscuring individual cellular contributions even with deconvolution.^38^ Emerging platforms, e.g., Visium HD (2 μm spot), achieve single-cell resolution and should be adopted in future studies. Fourth, this study represents a static snapshot from a single healthy, young male mouse and therefore does not directly evaluate reproducibility across animals or the ability of the ST framework to discriminate genetically modified or treatment-perturbed mice from their respective controls. Accordingly, the results should be interpreted as exploratory and cannot reflect biological variability related to sex, age, developmental stage, or disease progression. The relatively modest communication between skeletal muscle and bone in this study likely reflect a homeostatic state of bone-muscle crosstalk, and such crosstalk may be more prominent under non-homeostatic conditions, such as aging, injury, or diseases, when osteokine/myokine signaling and adipocyte activity are elevated.^70,119,120^ Future studies incorporating larger, diverse, and biologically replicated cohorts, together with controlled genetic or pharmacologic perturbations, will be necessary to validate and extend our findings, assess reproducibility, quantify differential spatial signaling, and establish the discriminatory power of this approach.

In conclusion, we present an initial spatially resolved transcriptome-wide map of cell-cell communication network within mouse femur and adjacent skeletal muscle, advancing mechanistic understanding of bone-muscle crosstalk and laying the groundwork for future studies in musculoskeletal disease.

## Materials and methods

### Animal

A 3-week-old male C57BL/6 mouse was obtained from Charles River Laboratory (Wilmington, USA) and housed under pathogen-free conditions in an individually ventilated cage at the Tulane University Animal Center for 2 weeks before experimentation. At 5 weeks of age, the mouse was euthanized for tissue collection. All procedures, including health monitoring and feeding, were conducted under sterile conditions. The mouse had ad libitum access to a standard cereal-based diet (Teklad 8604) and sterilized water, with a 12-h light/dark cycle maintained throughout. All animal experiments were approved by the Tulane University Institutional Animal Care and Use Committee.

### Tissue harvest and histology preparation

The right femur with attached skeletal muscle was harvested immediately after euthanasia, bisected, and fixed in 10% buffered formalin at 4 °C overnight. The sample was decalcified in 14% EDTA (pH 8.0) for 2 weeks with solution changes every 1–2 days, then paraffin embedded. Longitudinal 4 μm sections including the femoral head were prepared. Sample with DV200 ≥ 30% was used for further analysis. Libraries were generated using the Visium CytAssist Spatial Gene Expression for FFPE Kit (10x Genomics), which profiles transcripts within ~55 μm diameter spots. Library preparation followed protocol CG000520, and the section was processed according to the Visium CytAssist Tissue Preparation Guide (CG000518). After deparaffinization, the section was H&E-stained, imaged at 20× magnification (NanoZoomer S60v2MD, Hamamatsu), and then decoverslipped, destained, decrosslinked, and hybridized with the Visium Mouse Transcriptome Probe Set v1.0. Barcoded libraries were amplified, indexed, and sequenced on an Illumina NextSeq 2000 (2 × 96 bp), yielding > 100 million reads in this sample.

### Visium data processing and analysis

Demultiplexing and alignment were performed using 10x Genomics Space Ranger (v2.1.1), followed by image registration to H&E-stained section. The resulting feature matrix (HDF5 format) was imported into Seurat (v5.1.0) for quality control and downstream analysis. Data was normalized using SCTransform, and dimensionality reduction was conducted via principal component analysis (PCA). Unsupervised clustering was executed utilizing the Louvain algorithm (resolution = 0.5).

### Human subjects

Human femoral head specimens were collected from three patients (ages 57–78) undergoing osteoarthritic hip hemiarthroplasty at Tulane Hospital under approval from the Tulane University Institutional Review Boards. All participants provided written informed consent prior to tissue collection.

### scRNA-seq and data analysis

Mouse scRNA-seq data (GSE128423), enriched for bone marrow stromal cells from femur and tibia bones of four 8–10-week-old male C57BL/6 mice,^95^ were obtained from the Gene Expression Omnibus (GEO) database (https://www.ncbi.nlm.nih.gov/). Human bone single-cell suspensions were prepared from femoral head biopsies following a published protocol.^121^ Briefly, bone fragments (0.5–1 cm^3^) were digested twice with type II collagenase (1 mg/mL, 37 °C, 30 + 60 min), filtered (70 µm), treated with RBC lysis buffer, and depleted of hematopoietic (CD45^+^) cells using CD45 MicroBeads (Miltenyi Biotec). Cell suspensions (>70% viability) were processed on the 10× Genomics Chromium platform with the Chromium Fixed RNA Kit and sequenced on Illumina NextSeq 2000 (P3 100-cycle kit; ~6 500 cells/sample, ~50 000 reads/cell). Raw data were processed with Cell Ranger v6.1.2 for alignment and quantification. Downstream analyses in Seurat (v5.1.0) included quality control, log-normalization, PCA-based dimensionality reduction, Louvain clustering, and UMAP visualization. Cell types were annotated based on canonical marker gene expression.

### Histology-guided Visium spot region annotation

Anatomical compartments were manually annotated in the 10x Genomics Loupe Browser (v5.0.1) using the CLOUPE file generated by Space Ranger (v2.1.1). The H&E-registered image was used to delineate four regions based on standard histomorphological criteria^122,123^: cortical bone (compact lamellar cortex bounded by periosteal and endosteal surfaces), trabecular bone (lattice-like trabeculae interdigitating with marrow spaces), bone marrow (hematopoietic stroma and sinusoids with scattered adipocytes), and skeletal muscle (organized myofiber fascicles with visible perimysium and endomysium adjacent to bone). Each Visium spot was assigned to the region covering >50% of its area (“majority-area rule”) to prevent duplicate labeling while acknowledging that boundary spots may contain mixed signals. Annotation fidelity was validated by examining expression of canonical markers, such as Col1a1/Postn (cortical bone), Sp7/Runx2 (trabecular bone), Mki67/Ibsp (bone marrow), and Myh4/Mylpf (skeletal muscle). These markers were selected from ST gene list based on their known biological roles in bone and skeletal muscle. Gene compartmental enrichment score was quantified as the log_2_fold-change of expression in each region relative to the mean across other regions; positive scores indicate enrichment, and negative scores indicate depletion.

### Multiplex immunostaining of bone and skeletal muscle sections

Sequential multiplex immunostaining was performed using the COMET™ system (Lunaphore Technologies) according to the manufacturer’s instructions. Primary antibodies included anti-Runx2, anti-Mylpf, anti-Sdc4, anti-Col1a1, anti-Col1a2, anti-Col4a1, anti-Tnxb, anti-CD11b, and anti-CD44. Each automated staining cycle involved antigen retrieval, blocking, incubation with primary and HRP-conjugated secondary antibodies, and fluorophore deposition via tyramide signal amplification. Following each cycle, oxidative bleaching was applied to remove residual fluorescence before the next staining round. Nuclei were counterstained with DAPI and mounted using ProLong™ Gold Antifade Mountant. Multichannel fluorescence images were acquired on a Nikon Eclipse Ti2 confocal microscope, and colocalization of representative L-R pairs was qualitatively assessed using StrataQuest and ImageJ software.

### Differential gene expression and functional enrichment analysis

Differentially expressed genes between skeletal muscle and bone/bone marrow were identified using R package DEsingle (v1.18.0).^124^ Significance was determined based on an FDR (Benjamini-Hochberg) < 0.05 and an absolute log_2_fold-change >1. Functional enrichment analysis of the identified gene sets was performed using ClueGO (v2.5.9)^125^ under default settings. A Bonferroni-corrected *P* < 0.05 was employed to define statistically significant enrichment terms.

### Cell type deconvolution analysis

Deconvolution methods are critical for estimating cell-type composition from Visium ST. Reference-based approaches, such as CARD^36^ and Cell2location,^37^ leverage scRNA-seq references but their accuracy often depends on the availability of high-quality, tissue-matched data and can be confounded by batch effects.^36,37^ To overcome these limitations, we employed SMART v1.0,^38^ a reference-free, marker-gene-assisted deconvolution approach based on semi-supervised topic modeling. SMART integrates curated cell-type markers into a probabilistic framework and applies constrained optimization to decompose mixed gene expression within each spot, thereby estimating cell-type proportions and reconstructing cell-type-specific expression profiles. By leveraging marker gene priors, SMART enhances both spatial resolution and biological interpretability.^38^ To build a comprehensive and biologically relevant marker panel, we curated genes from the Mouse Cell Atlas^44^ and CellMarker 2.0.^45^ From the Mouse Cell Atlas, 1 356 unique markers across 16 annotated cell types (adipocytes, B cells, basophils, endothelial cells, eosinophils, erythroid cells, fibroblasts, hematopoietic stem and progenitor cells, megakaryocytes, MSCs, monocyte/macrophage, myeloid cells, neutrophils, osteoblasts, T cells, skeletal muscle cells) relevant to bone/bone marrow and skeletal muscle were selected (*P* < 0.001, absolute log_2_fold-change >1), with a median of 87 genes per cell type. To enhance coverage, we integrated additional 379 experimentally validated markers from CellMarker 2.0, including 161 overlapped with the Mouse Cell Atlas, resulting in a final panel of 1 574 unique markers across 16 cell types, with a median of 109 genes per cell type. This marker panel was used to initialize topic priors, and cell-type proportions were optimized under a Dirichlet-multinomial framework, producing a normalized spot × cell-type composition matrix. For each spot, the dominant cell type was annotated by the highest estimated proportion of that cell type. We defined mixed-profile spots as those in which the second most abundant cell type exceeds 50% of the proportion of the dominant cell type.

### Intercellular communication analysis

Intercellular signaling networks were inferred using CellChat v2.0,^49^ which integrates curated L-R databases with probabilistic modeling of expression patterns to construct cell-cell communication networks. To minimize noise, CellChat identifies differentially expressed signaling genes across cell groups (Wilcoxon test, *α* = 0.05) and computes robust group-level expression averages for each signaling component using *trimean* statistics. Communication probabilities are then estimated under a mass-action framework that incorporates multimeric ligands and receptors, membrane-bound co-factors, and Hill-type saturation effects to quantify the relative strength of each L-R interaction. These values represent interaction strengths rather than statistical probabilities. Statistical significance of each L-R pair is assessed by permutation testing (*M* = 100 randomizations), and L-R pairs with *P* < 0.05 are considered significant. For each pair of cell groups, CellChat reports (i) the number of significant interactions and (ii) the total communication strength, defined as the sum of probabilities of all significant L-R pairs connecting the two groups. To identify coordinated signaling programs, the three-dimensional communication tensor *P*_*i, j, k*_ (sender *i* × receiver *j* × L-R/pathway *k*) was summarized along sender or receiver dimensions to generate outgoing and incoming communication matrices. Each matrix was decomposed using non-negative matrix factorization to extract latent factors representing dominant signaling patterns. For visualization, cell- and pathway-loading matrices were normalized to the (0, 1) range, and values below 0.5 were set to zero to highlight enriched associations. In pathway-specific heatmaps, the outgoing and incoming signaling strengths were further rescaled within each pathway across all cell groups, which ensure comparability within each pathway while preventing globally dominant pathways from overshadowing others.

## Supplementary information

Supplementary Materials

## Data Availability

The mouse scRNA-seq data that support the findings of this study obtained from GEO with the accession number GSE128423 (https://www.ncbi.nlm.nih.gov/geo/query/acc.cgi?acc=GSE128423). All other relevant data from this study are available from the corresponding authors upon reasonable request.
