# Supplementary material for: Decoding cellular communication networks and signaling pathways in bone, skeletal muscle, and bone-muscle crosstalk through spatial transcriptomics in a young male mouse
Source: Bone Res. 2026 May 19;14:55. doi: 10.1038/s41413-026-00520-w (PMC13187134; doi:10.1038/s41413-026-00520-w)
Supplement: Supplementary file 1 — Supplementary Materials [file 41413_2026_520_MOESM1_ESM.docx]

**Supplementary Materials**

**Decoding cellular communication networks and signaling pathways in bone, skeletal muscle, and bone-muscle crosstalk through spatial transcriptomics in a young male mouse**

Runing Title: Decoding bone-muscle crosstalk via spatial transcriptomics

Chuan Qiu ^1^, Yisu Li ^2^, Yun Gong ^1^, William Sherman ^3^, Di Tian ^4^, Weiqiang Lin ^1^, Zehui Pan ^1^, Boluwatife Afolabi ^1^, Vivek Thumbigere ^5^, Kuanjui Su ^1^, Jeffrey Deng ^6^, Yuwei Hou ^1^,

Shashank Mungasavalli Gnanesh ^1^, Zhe Luo ^1^, Qing Tian ^1^, Fernando Sanchez ^3^, Yiping Chen ^2^,

Hui Shen ^1^, Hong-Wen Deng ^1*^

1. Tulane Center for Biomedical Informatics and Genomics, Deming Department of Medicine, School of Medicine, Tulane University, New Orleans, LA 70112, USA
2. Department of Cell and Molecular Biology, School of Science and Engineering, Tulane University, New Orleans, LA 70118, USA
3. Department of Orthopedic Surgery, School of Medicine, Tulane University, New Orleans, LA 70112, USA
4. Department of Pathology and Laboratory Medicine, School of Medicine, Tulane University, New Orleans, LA 70112, USA
5. Division of Periodontology, Department of Advanced Oral Sciences & Therapeutics, School of Dentistry, University of Maryland, Baltimore, MD 21201, USA
6. Geisel School of Medicine, Dartmouth College, Hanover, NH 03755, USA


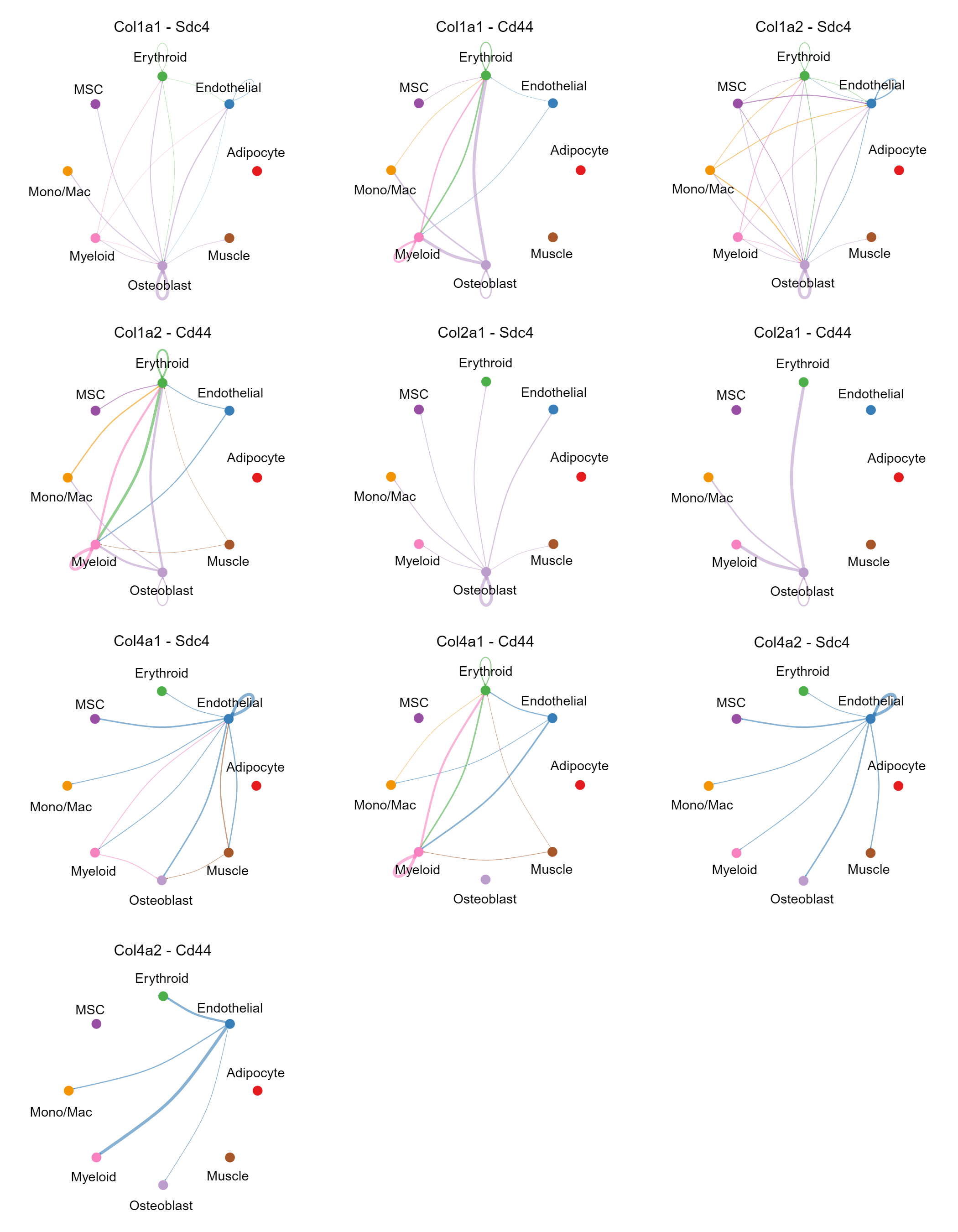


**Fig. S1** Cell-cell communications mediated via 10 L-R pairs within COLLAGEN signaling pathway.

**
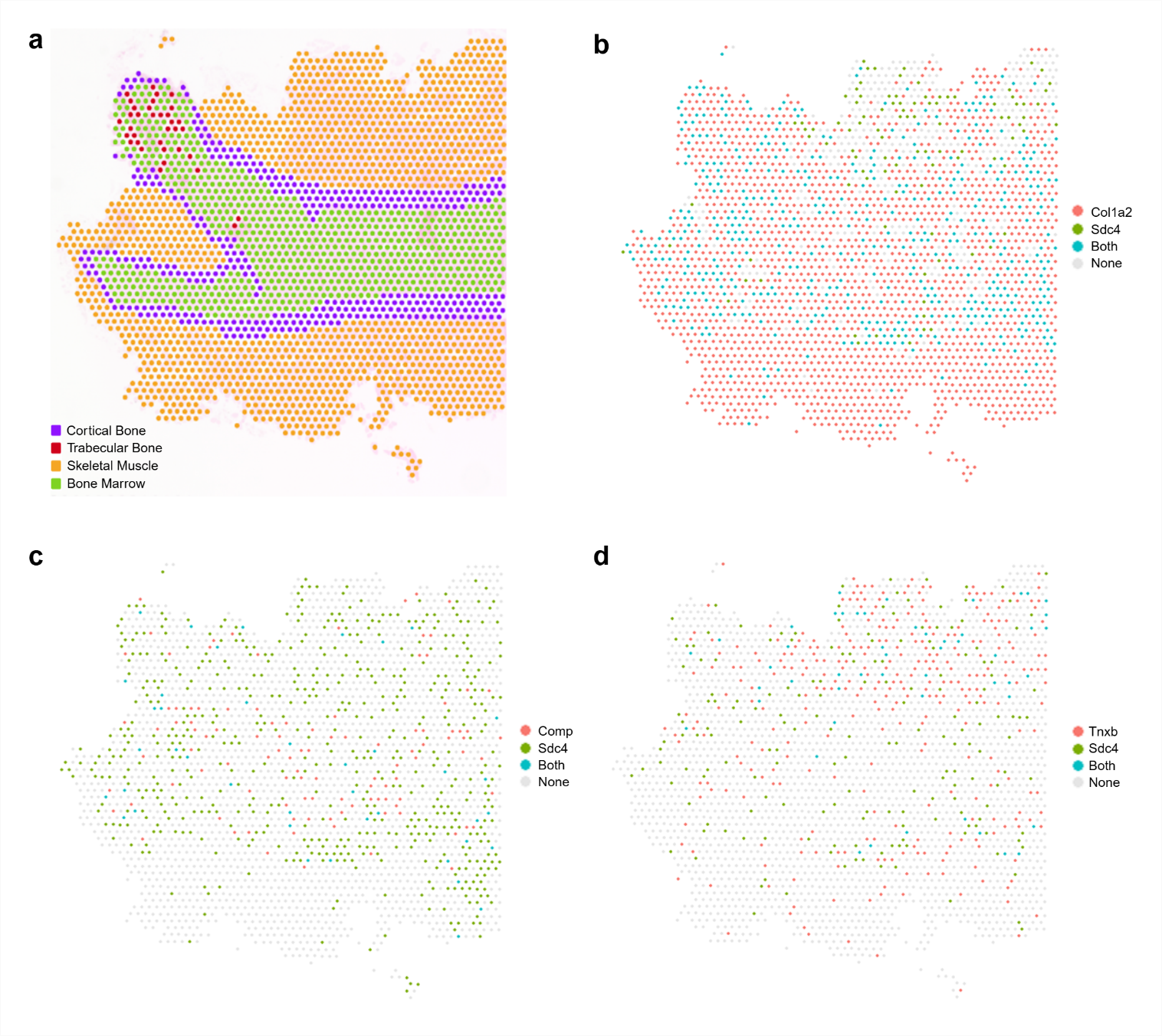
**

**Fig. S2** Visualization of histology-guided annotation with spatial distribution of L-R signaling networks. **a** H&E image with manual region annotations in Loupe Browser v5.0.1 (purple: cortical bone; red: trabecular bone; green: marrow; yellow: skeletal muscle). **b-d** Diagrams show the spatial localization of cell-cell communications via **b**) Col1a2-Sdc4 L-R pair in COLLAGEN pathway, **c)** Comp-Sdc4 L-R pair in THBS pathway, and **d)** Tnxb-Sdc4 L-R pair in TENASCIN pathway, respectively. This integrated view enables direct comparison of the spatial distribution of L-R interactions relative to the annotated musculoskeletal compartments.


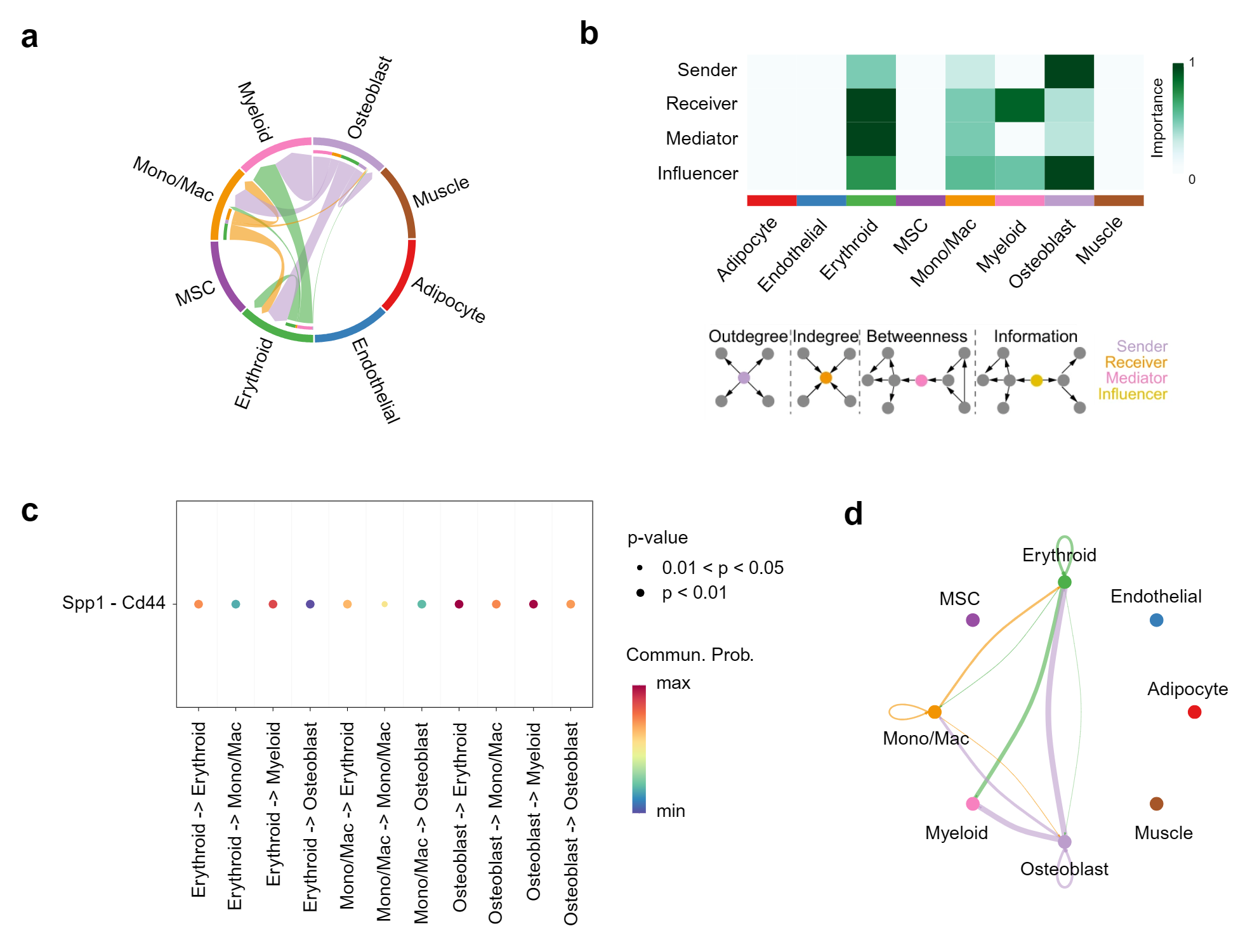


**Fig. S3** Cell-cell communications and their corresponding L-R interaction pairs in SPP1 signaling pathway. **a** A chord diagram shows the cell-cell communications in SPP1 signaling pathway. **b** Network centrality of the SPP1 signaling pathway. The darker colors indicate greater importance. **c** Dot plot shows the significant L-R pairs across various cell types in SPP1 signaling pathway. **d** Cell-cell communication mediated by a Spp1-Cd44 L-R pair in SPP1 signaling pathway.


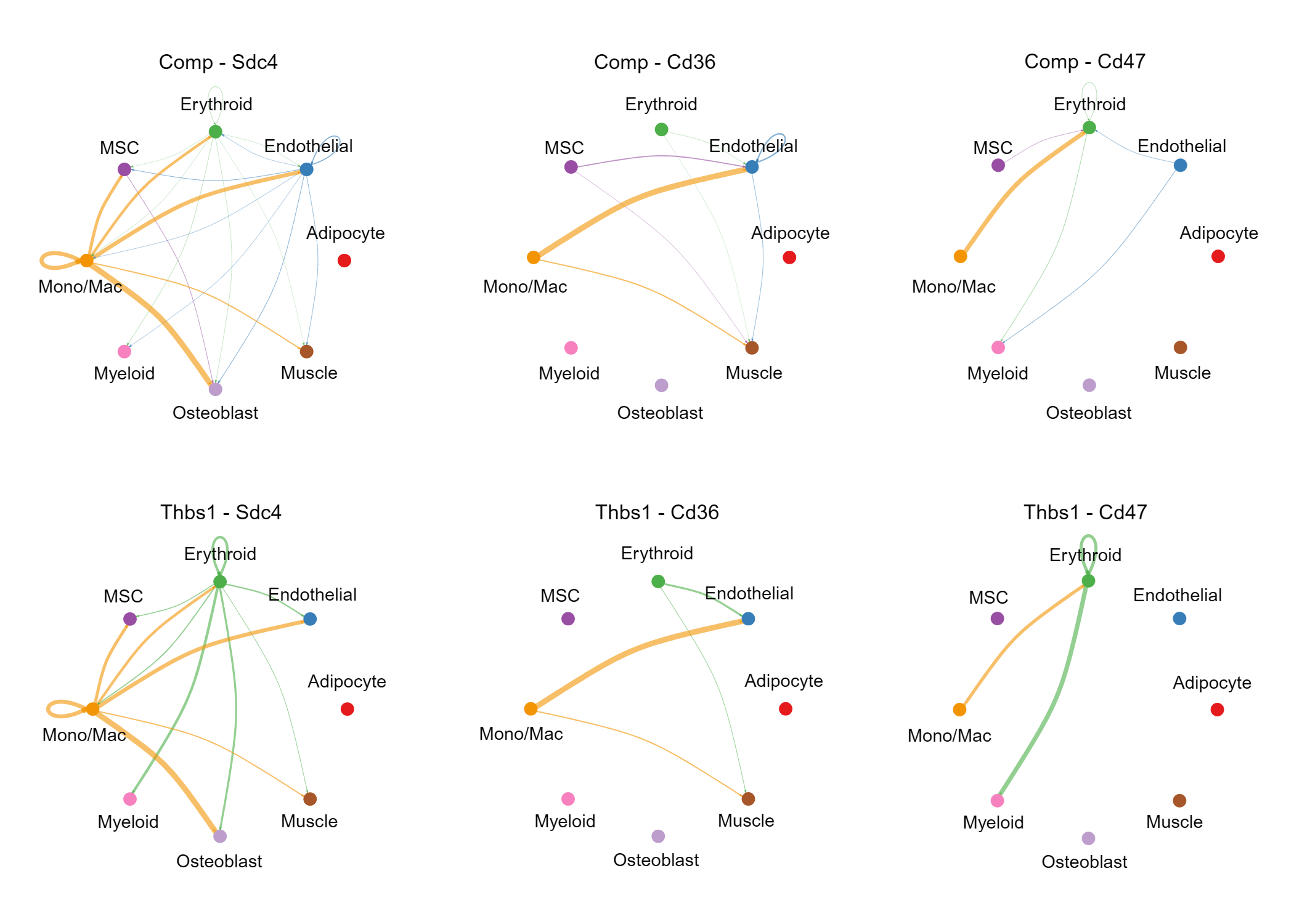


**Fig. S4** Cell-cell communications mediated via 6 L-R pairs within THBS signaling pathway.


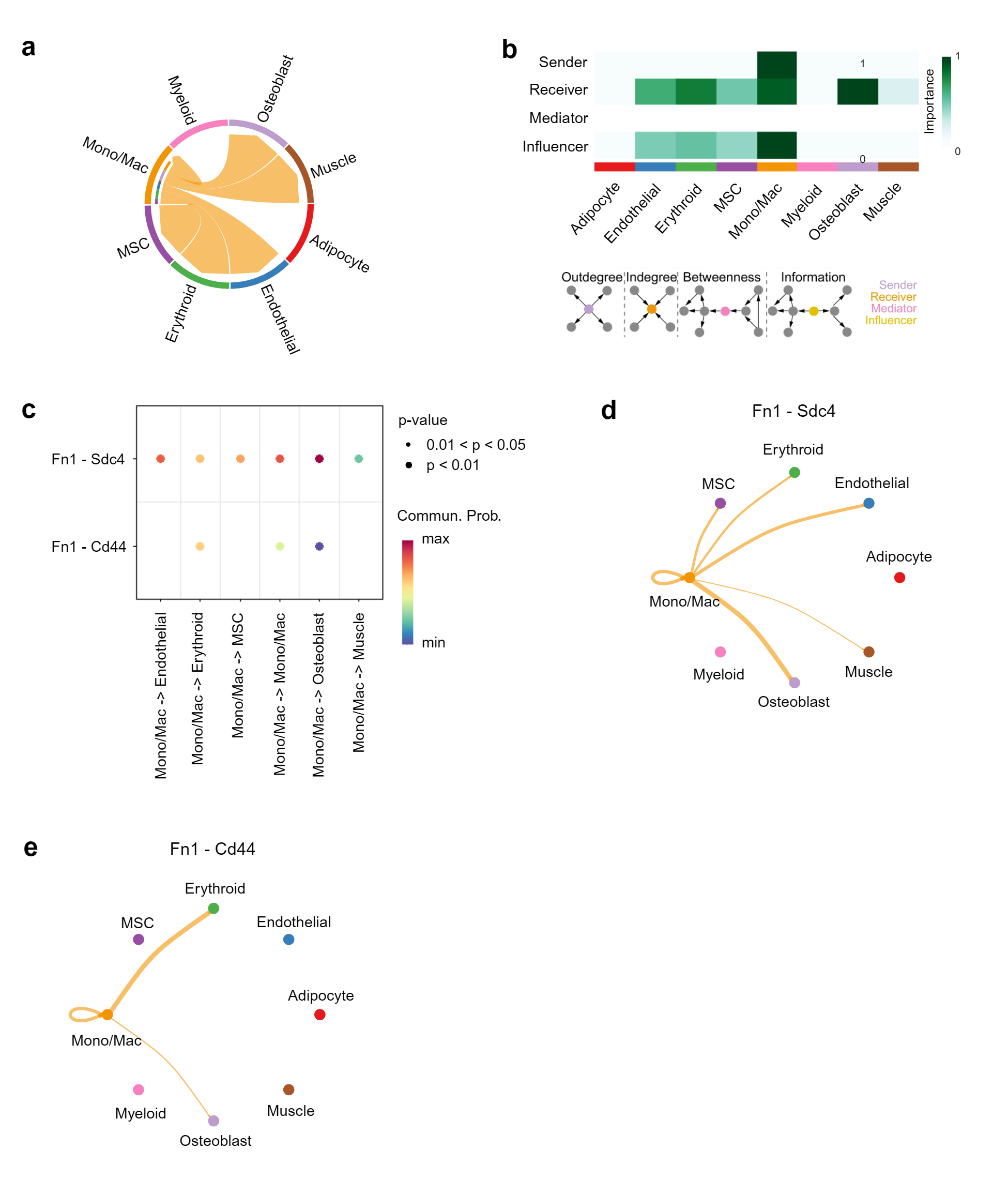


**Fig. S5** Cell-cell communications and their corresponding L-R interaction pairs in FN1 signaling pathway. **a** A chord diagram shows the cell-cell communications in FN1 pathway. **b** Network centrality of the FN1 signaling pathway. The darker colors indicate greater importance. **c** Dot plot shows the significant L-R pairs across various cell types in FN1 signaling pathway. **d-e** Cell-cell communication is mediated by Fn1-Sdc4 (**d**) and Fn1-Cd44 (**e**) L-R pairs in FN1 signaling pathway.

**
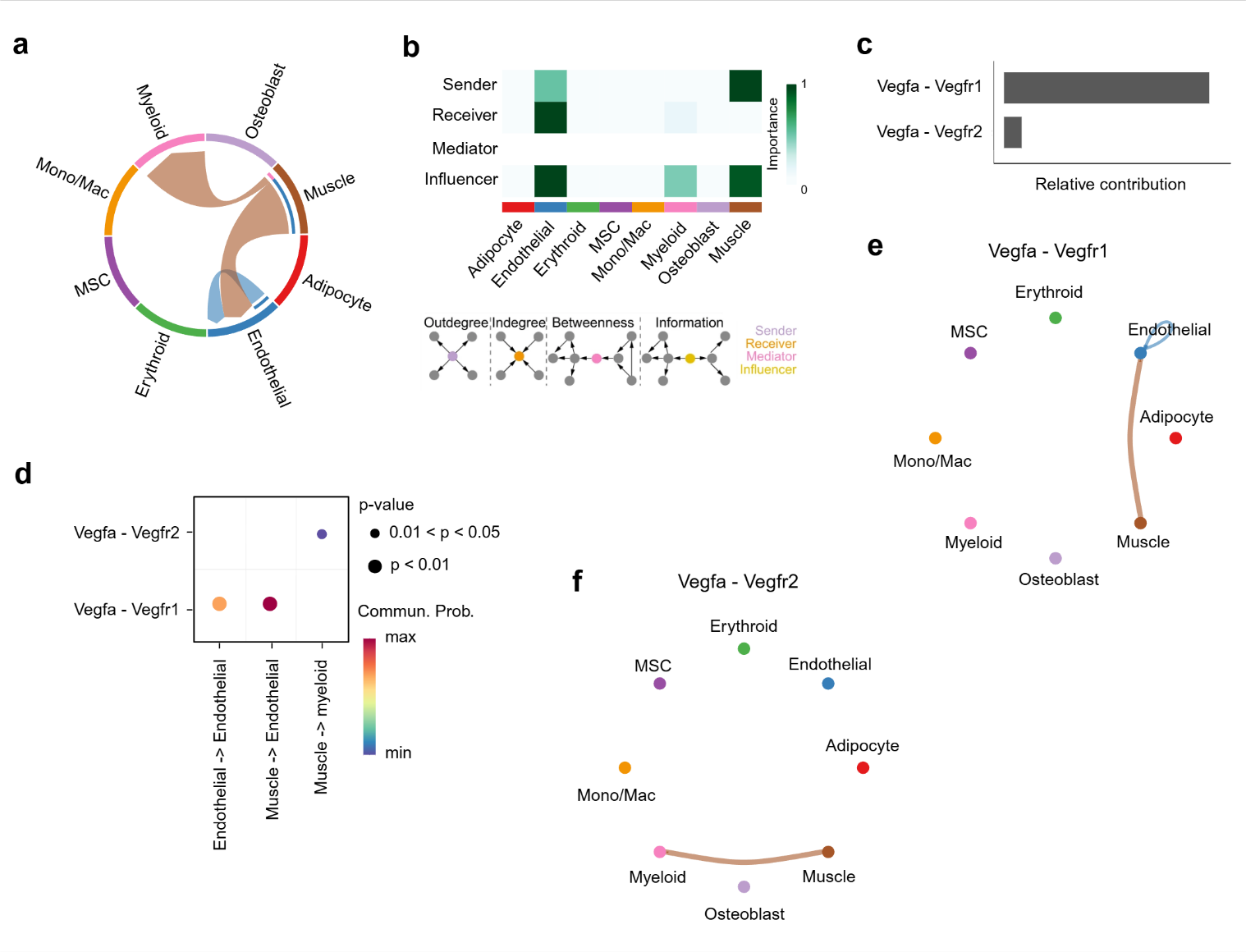
**

**Fig. S6** Cell-cell communications and their corresponding L-R interaction pairs in VEGF signaling pathway. **a** A chord diagram showing cell-cell communications in VEGF pathway. **b** Network centrality of the VEGF signaling pathway. The darker colors indicate greater importance. **c** Relative contributions of L-R pairs within the pathway. **d** Dot plot of significant L-R pairs across various cell types in VEGF signaling pathway (color = communication probability; dot size = significance). **e-f** Cell-cell communication mediated by (**e**) Vegfa-Vegfr1 and (**f**) Vegfa-Vegfr2 L-R pairs in VEGF signaling.


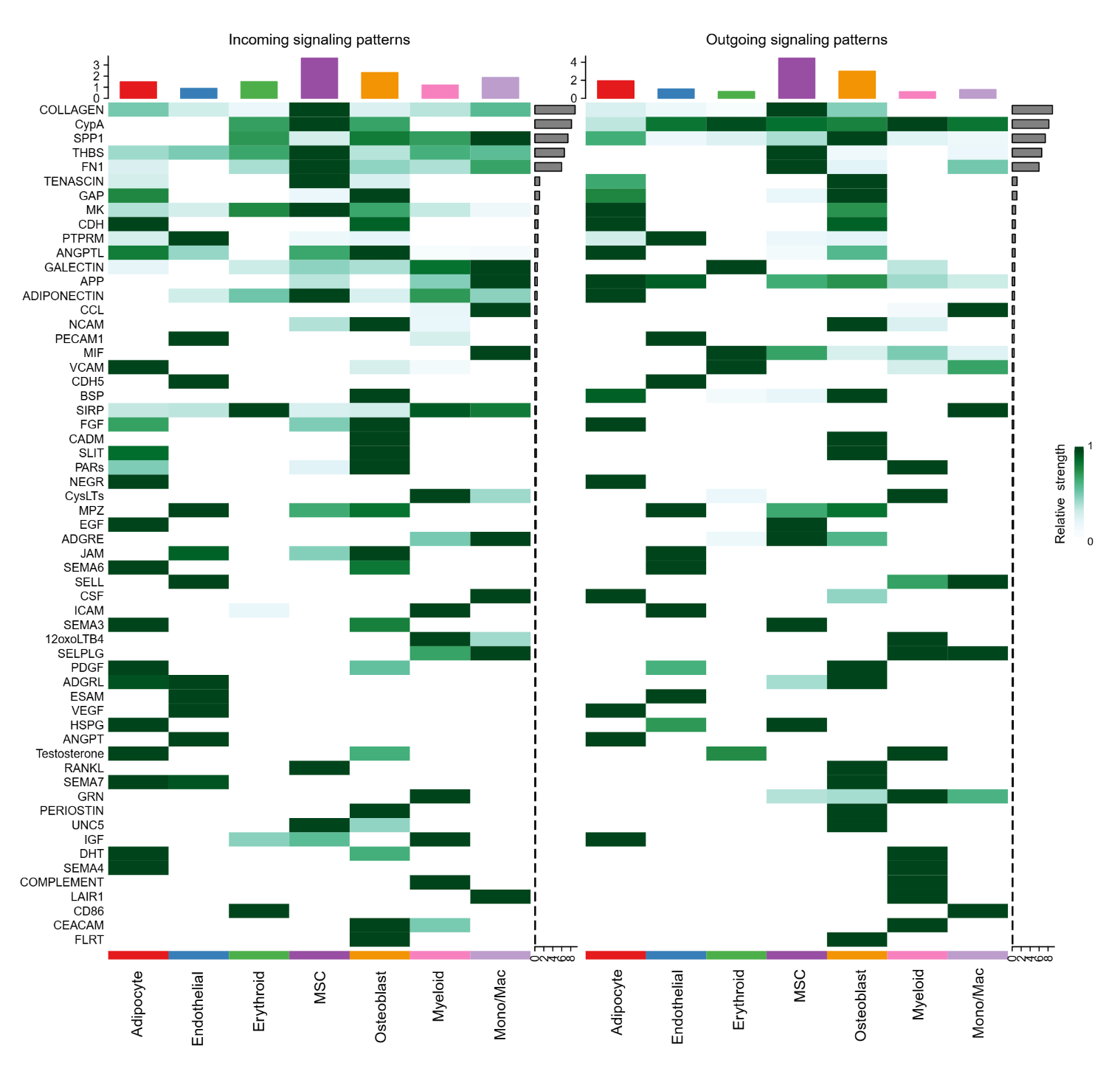


**Fig. S7** Outgoing and incoming communication patterns in mice bone scRNA-seq dataset. Heatmaps showing pathways contributing to incoming (left) and outgoing (right) signals of each cell group. The “Relative strength” scale (0-1) reflects row-scaled normalization for each pathway across all cell groups. The top-colored bar: total signaling strength of each cell group. The right grey bar: total strength of each pathway.


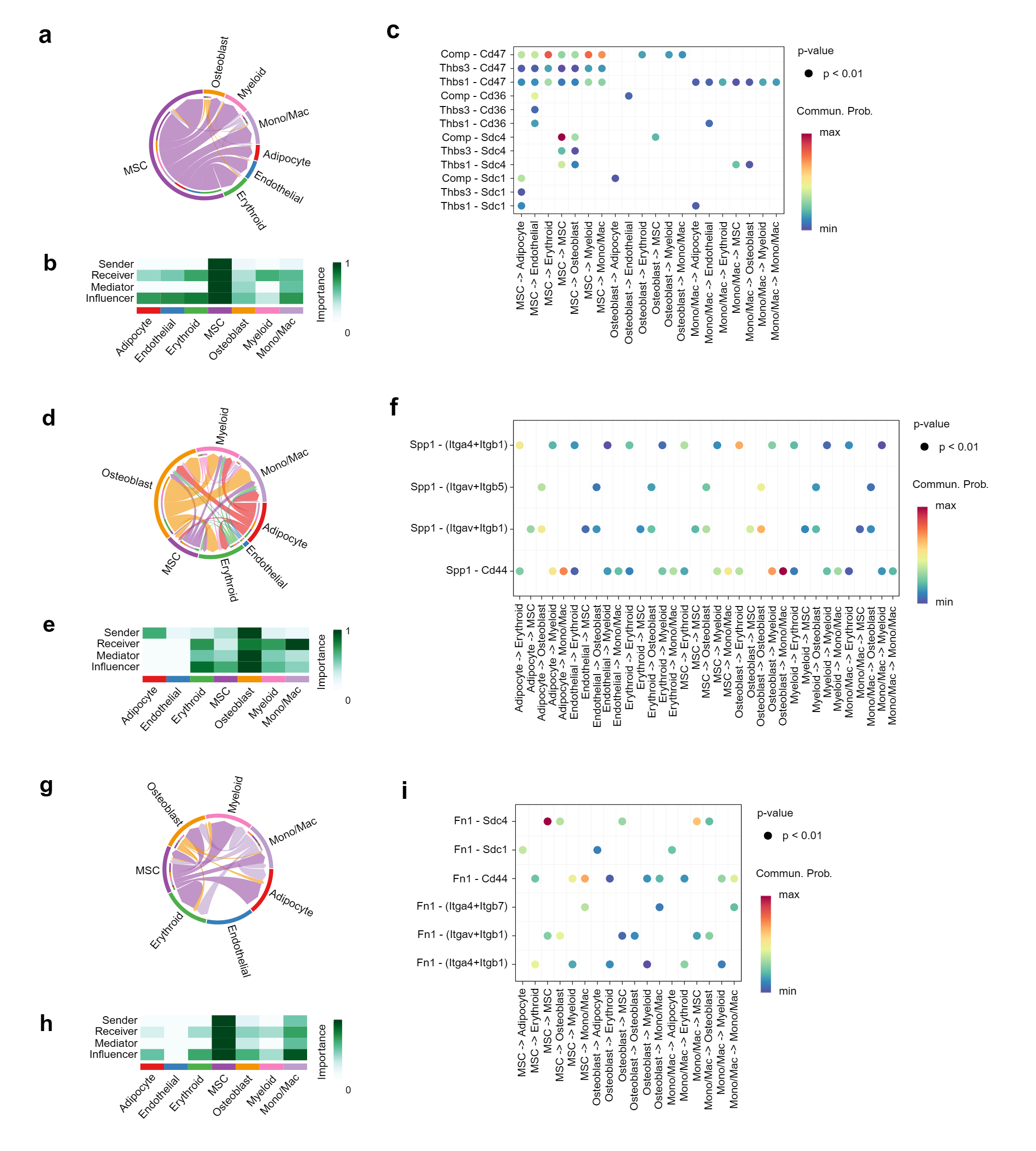


**Fig. S8** Cell-cell communications in mice bone scRNA-seq dataset and their corresponding L-R interaction pairs in THBS, SPP1, and FN1 signaling pathways. **a, d, g** The chord diagrams showing cell-cell communications in each pathway. **b, e, h** Network centrality roles computed on the pathway-specific weighted-directed network: senders (out-degree), receivers (in-degree), mediators (flow betweenness), influencers (information centrality). Darker color indicates greater role magnitude. **c, f, i** Dot plot of significant L-R pairs across various cell types in each signaling pathway. For each L-R pair, color represents the communication probability, and dot size indicates the statistical significance of the interaction within each cell-cell communication pair.


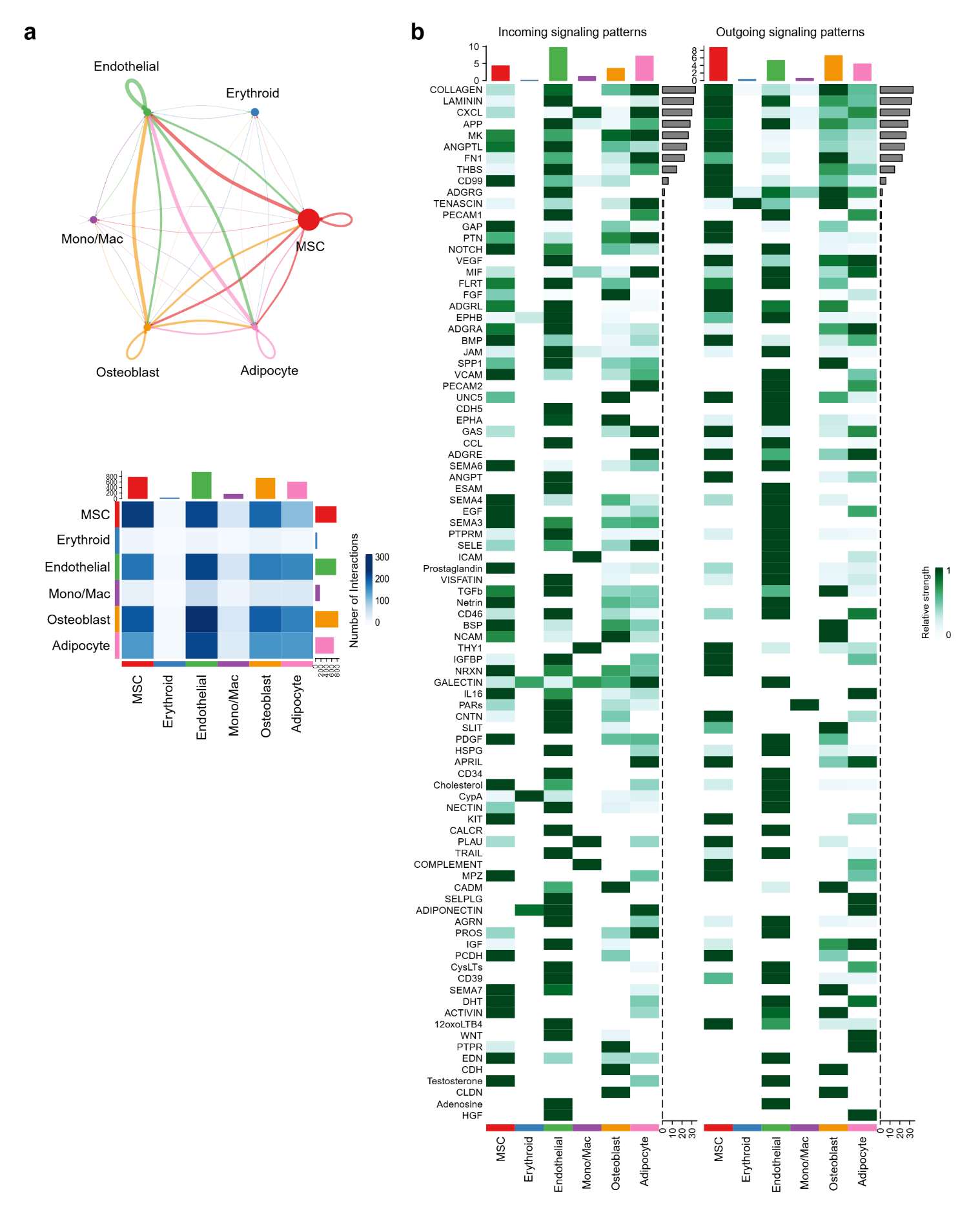


**Fig. S9** The cell-cell communication pattern in human bone scRNA-seq data. **a** Chord diagram (up) depicting the number of inferred interactions between each pair of cell populations. Heatmap (down) showing interaction counts between sending (rows) and receiving (columns) cell population. Higher intensity indicates greater number between the sending cluster and the receiving cluster. The numbers of interactions were computed by CellChat based on probabilistic modeling of L-R expression, with significance determined by permutation testing (p < 0.05). **b** Heatmaps showing pathways contributing to incoming (left) and outgoing (right) signals of each cell group. The “Relative strength” scale (0-1) reflects row-scaled normalization for each pathway across all cell groups. The top-colored bar: total signaling strength of each cell group. The right grey bar: total strength of each pathway.

**Fig. S10** Cell-cell communications in human bone scRNA-seq dataset and their corresponding L-R interaction pairs in COLLAGEN and THBS signaling pathways. **a, d** The chord diagrams showing cell-cell communications in (**a**) COLLAGEN and (**d**) THBS signaling pathway. **b, e** Network centrality roles in (**b**) COLLAGEN and (**e**) THBS signaling pathway, computed on the pathway-specific weighted-directed network: senders (out-degree), receivers (in-degree), mediators (flow betweenness), influencers (information centrality). Darker color indicates greater role magnitude. **c, f,** Dot plot of significant L-R pairs across various cell types in (**c**) COLLAGEN and (**f**) THBS signaling pathway. For each L-R pair, color represents the communication probability, and dot size indicates the statistical significance of the interaction within each cell-cell communication pair.


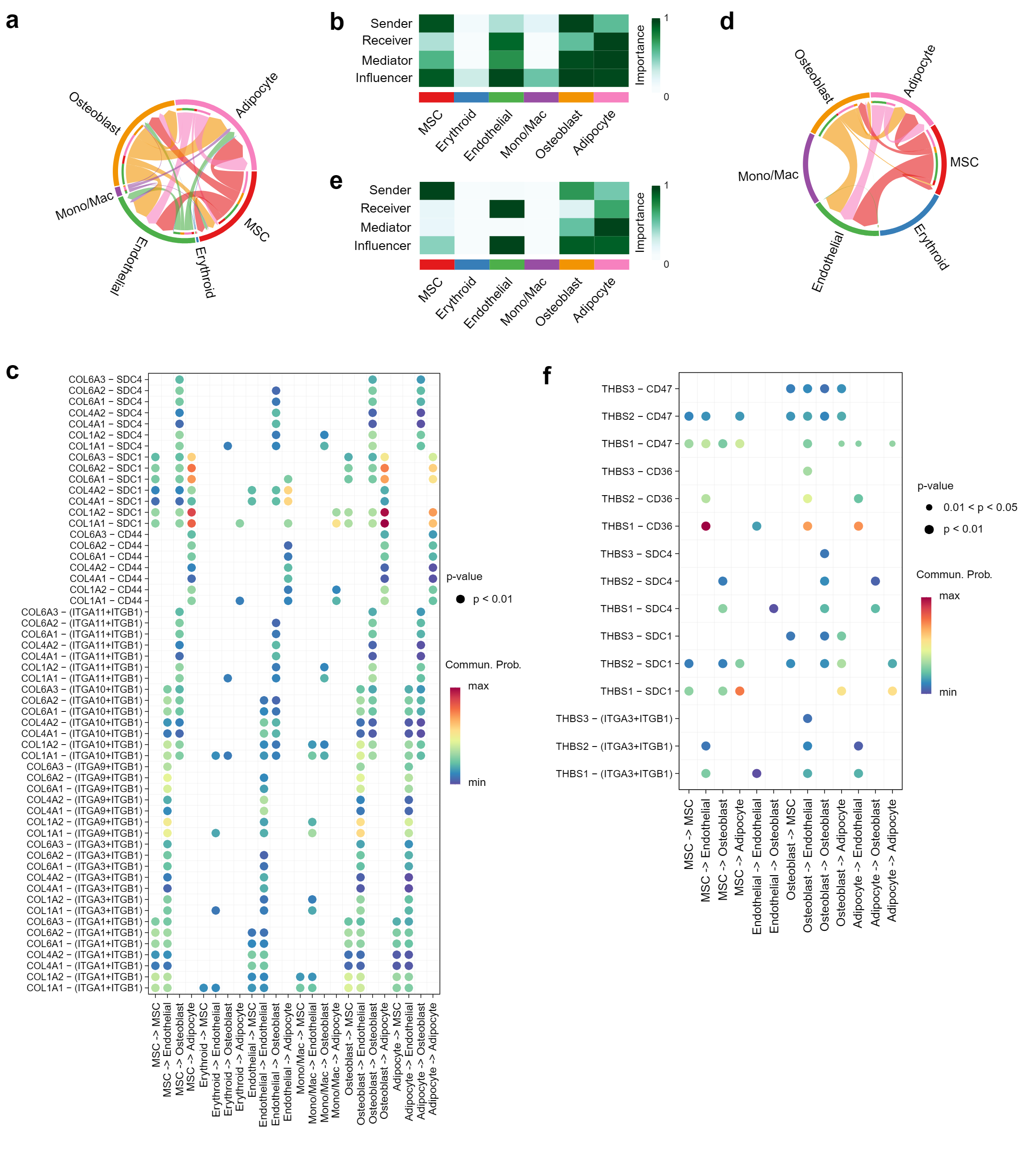

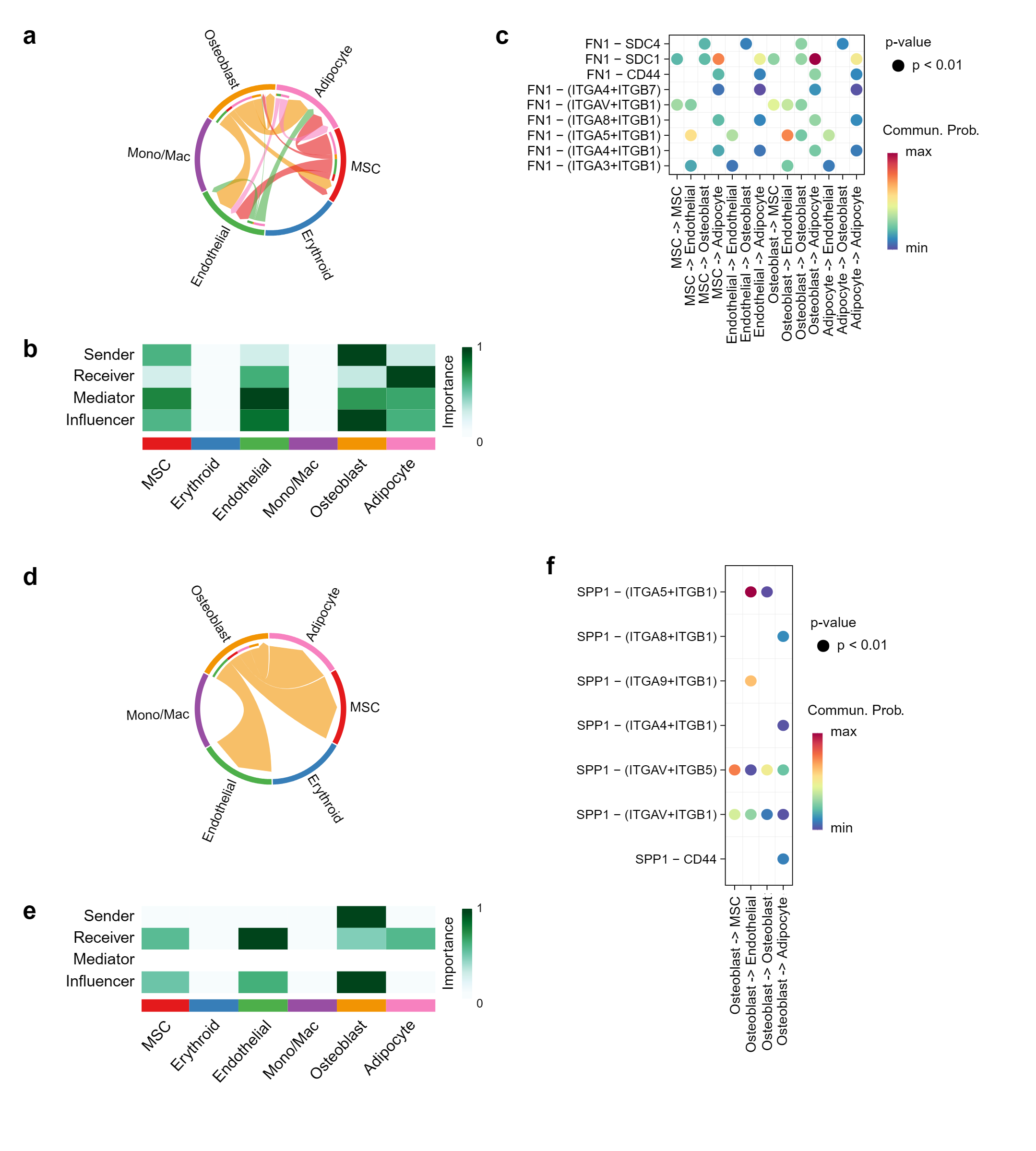


**Fig. S11** Cell-cell communications in human bone scRNA-seq dataset and their corresponding L-R interaction pairs in FN1 and SPP1 signaling pathways. **a, d** The chord diagrams showing cell-cell communications in (**a**) FN1 and (**d**) SPP1 signaling pathway. **b, e** Network centrality roles of (**b**) FN1 and (**e**) SPP1 signaling pathway, computed on the pathway-specific weighted-directed network: senders (out-degree), receivers (in-degree), mediators (flow betweenness), influencers (information centrality). Darker color indicates greater role magnitude. **c, f,** Dot plot of significant L-R pairs across various cell types in (**c**) FN1 and (**f**) SPP1 signaling pathway. For each L-R pair, color represents the communication probability, and dot size indicates the statistical significance of the interaction within each cell-cell communication pair.


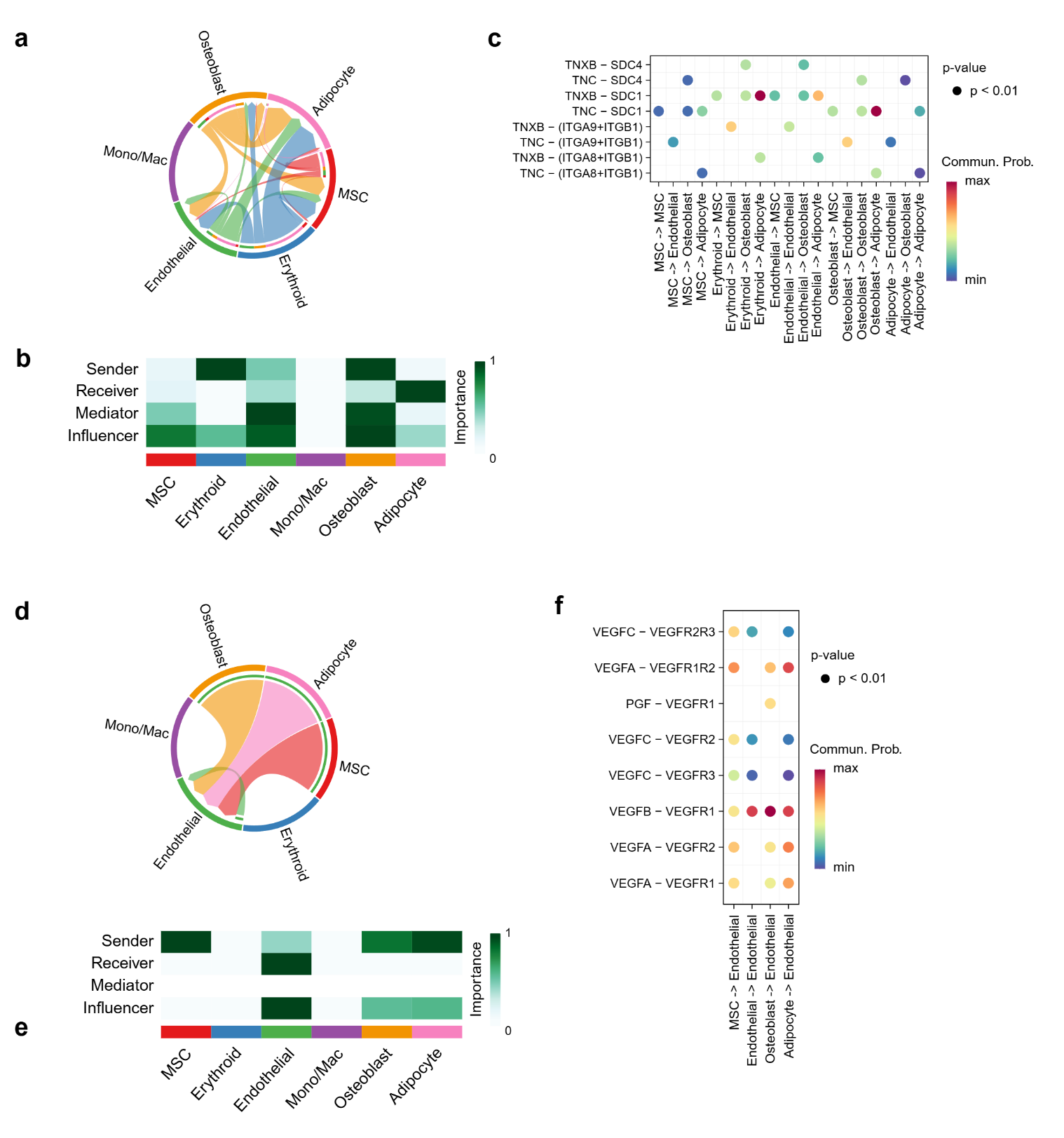


**Fig. S12** Cell-cell communications in human bone scRNA-seq dataset and their corresponding L-R interaction pairs in TENASCIN and VEGF signaling pathways. **a, d** The chord diagrams showing cell-cell communications in (**a**) TENASCIN and (**d**) VEGF signaling pathway. **b, e** Network centrality roles of (**b**) TENASCIN and (**e**) VEGF signaling pathway, computed on the pathway-specific weighted-directed network: senders (out-degree), receivers (in-degree), mediators (flow betweenness), influencers (information centrality). Darker color indicates greater role magnitude. **c, f,** Dot plot of significant L-R pairs across various cell types in (**c**) TENASCIN and (**f**) VEGF signaling pathway. For each L-R pair, color represents the communication probability, and dot size indicates the statistical significance of the interaction within each cell-cell communication pair.


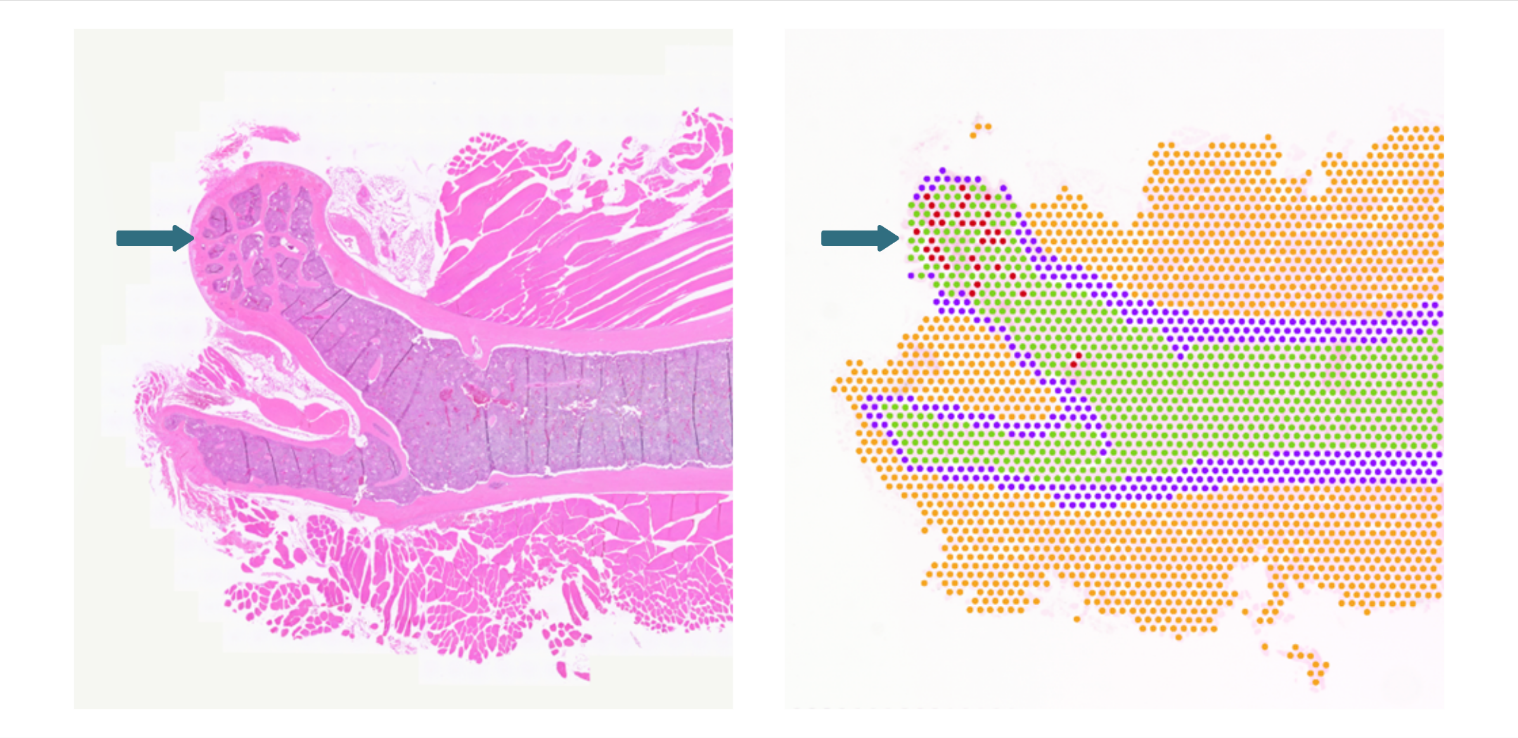


**Fig. S13** Partial bone detachment during Visium CytAssist slide preparation. Due to the high density and mineralization of bone, the bone detachment issue may affect spatial continuity and mapping accuracy, particularly in cortical bone at femoral head (arrow).


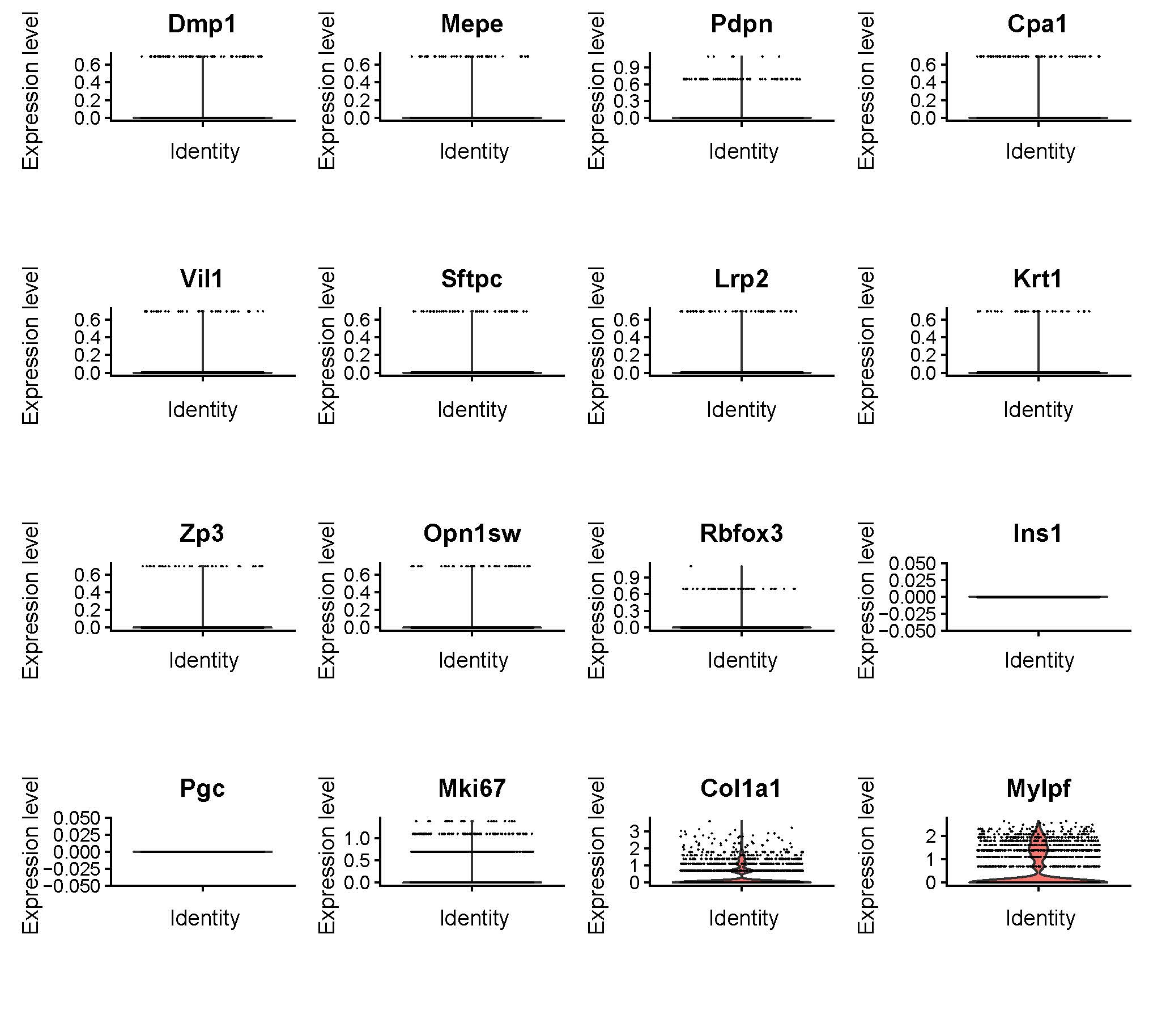


**Fig. S14** Gene expression pattern of osteocyte markers of Dmp1, Mepe, and Pdpn. The expression levels of Dmp1 and Mepe were remarkably similar to that of known marker genes for other tissues (not expected to be detected in bone-muscle sample), such as Cpa1 for pancreas, Vil1 for intestine/colon, Sftpc for alveolar type II cells, Lrp2 for kidney, Krt1 for skin, Zp3 for oocyte, Opn1sw for retina, Rbfox3 for neurons, Ins1 for Islet, and Pgc for stomach. Pdpn, which marks the early osteoblast-to-osteocyte transition, was expressed at relatively higher levels than Dmp1 and Mepe, consistent with previous reports of sparse early osteocyte signal in Visium ST (Xiao et al., Bone Research, 2023). Nevertheless, its expression levels remained fairly low and sparsely expressed compared with bone and skeletal muscle compartment markers in ST dataset, such as Mki67 (bone marrow), Col1a1 (cortical/trabecular bone), and Mylpf (skeletal muscle) in our dataset.

**Table S1**. Gene compartmental enrichment analysis for the representative compartment genes

| Sample | Postn | Col1a1 | Sp7 | Runx2 | Ptprc | Mki67 | Ibsp | Myh4 | Mylpf |
| --- | --- | --- | --- | --- | --- | --- | --- | --- | --- |
| Cortical Bone | 0.56 | 0.45 | -0.76 | -1.05 | -0.82 | -0.73 | -0.9 | 1.4 | 1.41 |
| Trabecular Bone | 0.27 | 0.38 | 0.97 | 1.11 | 0.28 | -0.23 | -2.94 | -2.28 | -2.18 |
| Bone Marrow | -0.11 | -0.43 | -0.29 | -0.07 | 0.28 | 0.6 | 1.21 | -1.88 | -2.41 |
| Muscle | -1.65 | -1.09 | -1.11 | -1.53 | -1.85 | -1.92 | -0.63 | 2.19 | 2.8 |

**Note:** Gene compartmental enrichment score was quantified as the log2 fold-change of expression in each region relative to the mean across other regions; positive scores indicate enrichment and negative scores indicate depletion.

**Table S2**. Key L-R genes in CellChat-inferred pathways

| Gene Symbol | Protein Name | Role in Pathway | Function (relevant to musculoskeletal biology) |
| --- | --- | --- | --- |
| Col1a1 | Collagen type I α1 chain | Ligand (COLLAGEN) | Major fibrillar collagen; provides tensile strength in bone, tendon, and muscle ECM [^1^](#_ENREF_1) |
| Col1a2 | Collagen type I α2 chain | Ligand (COLLAGEN) | Partner of Col1a1 in collagen I heterotrimer; regulates mineralization and ECM stability [^1^](#_ENREF_1) |
| Col2a1 | Collagen type II α1 chain | Ligand (COLLAGEN) | Major collagen in cartilage; maintains ECM integrity [^2^](#_ENREF_2) |
| Col4a1 | Collagen type IV α1 chain | Ligand (COLLAGEN) | Basement membrane component; supports vascular integrity and endothelial adhesion [^3^](#_ENREF_3) |
| Col4a2 | Collagen type IV α2 chain | Ligand (COLLAGEN) | Forms collagen IV heterotrimers; regulates angiogenesis and basement membrane structure [^3^](#_ENREF_3) |
| Sdc4 | Syndecan-4 | Receptor (COLLAGEN/THBS/FN1/TENASCIN) | Heparan sulfate proteoglycan receptor; mediates ECM–cell adhesion, mechanotransduction, and angiogenesis [^4^](#_ENREF_4) |
| Cd44 | CD44 | Receptor (COLLAGEN/SPP1/FN1) | Hyaluronan receptor; regulates cell adhesion, migration, and osteoclast activity [^5^](#_ENREF_5) |
| Spp1 | Osteopontin | Ligand (SPP1) | Secreted phosphoprotein; regulates bone remodeling, osteoclast adhesion, and muscle regeneration [^6^](#_ENREF_6) |
| Thbs1 | Thrombospondin-1 | Ligand (THBS) | ECM glycoprotein; modulates angiogenesis, inflammation, and osteoblast differentiation [^7^](#_ENREF_7) |
| Comp | Cartilage oligomeric matrix protein | Ligand (THBS) | ECM glycoprotein; stabilizes collagen fibrils; regulates chondrocyte/osteoblast function [^8^](#_ENREF_8) |
| Cd47 | CD47 (Integrin-associated protein) | Receptor (THBS) | Thrombospondin receptor; regulates apoptosis, immune evasion, and osteoclast fusion [^9^](#_ENREF_9) |
| Cd36 | CD36 (Scavenger receptor) | Receptor (THBS) | Binds thrombospondin and fatty acids; regulates angiogenesis and inflammation [^10^](#_ENREF_10) |
| Fn1 | Fibronectin 1 | Ligand (FN1) | ECM glycoprotein; mediates cell adhesion, migration, and wound healing [^11^](#_ENREF_11) |
| Vegfa | VEGF-A | Ligand (VEGF) | Key angiogenic factor; promotes endothelial proliferation and vascularization [^12^](#_ENREF_12) |
| Vegfr1 | VEGF receptor 1 | Receptor (VEGF) | VEGF receptor; regulates vascular development and inflammation [^13^](#_ENREF_13) |
| Vegfr2 | VEGF receptor 2 | Receptor (VEGF) | Primary VEGF receptor; mediates angiogenesis and endothelial survival [^14^](#_ENREF_14) |
| Tnxb | Tenascin-X | Ligand (TENASCIN) | ECM glycoprotein; regulates ECM integrity, muscle repair, and connective tissue homeostasis [^15^](#_ENREF_15) |

## References

1. Myllyharju, J. & Kivirikko, K.I. Collagens, modifying enzymes and their mutations in humans, flies and worms. *Trends Genet* **20**, 33-43 (2004).

2. Ricard-Blum, S. The collagen family. *Cold Spring Harb Perspect Biol* **3**, a004978 (2011).

3. Kalluri, R. Basement membranes: structure, assembly and role in tumour angiogenesis. *Nat Rev Cancer* **3**, 422-33 (2003).

4. Echtermeyer, F. *et al.* Delayed wound repair and impaired angiogenesis in mice lacking syndecan-4. *J Clin Invest* **107**, R9-R14 (2001).

5. Ponta, H., Sherman, L. & Herrlich, P.A. CD44: from adhesion molecules to signalling regulators. *Nat Rev Mol Cell Biol* **4**, 33-45 (2003).

6. Denhardt, D.T. & Guo, X. Osteopontin: a protein with diverse functions. *FASEB J* **7**, 1475-82 (1993).

7. Adams, J.C. & Lawler, J. The thrombospondins. *Cold Spring Harb Perspect Biol* **3**, a009712 (2011).

8. Briggs, M.D. & Chapman, K.L. Pseudoachondroplasia and multiple epiphyseal dysplasia: mutation review, molecular interactions, and genotype to phenotype correlations. *Hum Mutat* **19**, 465-78 (2002).

9. Oldenborg, P.A. CD47: A Cell Surface Glycoprotein Which Regulates Multiple Functions of Hematopoietic Cells in Health and Disease. *ISRN Hematol* **2013**, 614619 (2013).

10. Silverstein, R.L. & Febbraio, M. CD36, a scavenger receptor involved in immunity, metabolism, angiogenesis, and behavior. *Sci Signal* **2**, re3 (2009).

11. Pankov, R. & Yamada, K.M. Fibronectin at a glance. *J Cell Sci* **115**, 3861-3 (2002).

12. Ferrara, N. Vascular endothelial growth factor as a target for anticancer therapy. *Oncologist* **9 Suppl 1**, 2-10 (2004).

13. Shibuya, M. Vascular endothelial growth factor receptor-1 (VEGFR-1/Flt-1): a dual regulator for angiogenesis. *Angiogenesis* **9**, 225-30; discussion 231 (2006).

14. Olsson, A.K., Dimberg, A., Kreuger, J. & Claesson-Welsh, L. VEGF receptor signalling - in control of vascular function. *Nat Rev Mol Cell Biol* **7**, 359-71 (2006).

15. Mao, J.R. & Bristow, J. The Ehlers-Danlos syndrome: on beyond collagens. *J Clin Invest* **107**, 1063-9 (2001).
